# *In silico* analysis reveals widespread presence of three gene families, MAPK, MAPKK and MAPKKK, of the MAPK cascade from crop plants of *Solanaceae* in comparison to the distantly-related syntenic species from *Rubiaceae,* coffee

**DOI:** 10.7717/peerj.3255

**Published:** 2017-06-06

**Authors:** Hira Iftikhar, Nayab Naveed, Nasar Virk, Muhammad Faraz Bhatti, Fengming Song

**Affiliations:** 1 Atta-ur-Rahman School of Applied Biosciences (ASAB), National University of Sciences and Technology (NUST), Islamabad, Pakistan; 2 University Institute of Information Technology, PMAS-Arid Agriculture University, Rawalpindi, Pakistan; 3 National Key Laboratory for Rice Biology, Institute of Biotechnology, Zhejiang University, Hangzhou, China

**Keywords:** Mitogen-activated protein kinases, Maximum likelihood, Heat plot, Raf, MEKK, *Solanum lycopersicum*, *Solanum tuberosum*, *Solanum melongena*, *Capsicum annuum*, *Coffea canephora*

## Abstract

Mitogen-activated protein kinases (MAPKs) are an important family of genes which play roles in vital plant processes, and they also help in coping against various kinds of environmental stresses including abiotic as well as biotic factors. The advancement of genomics calls for the annotation, identification, and detailed processing of the essential gene families in plants in order to provide insights into the importance of their central roles as well as for providing the basis for making their growth vigorous even under stressed conditions and, ultimately, to benefit from them by foreseeing the potential threats to their growth. In the current study, MAPK, MAPKK, and MAPKKK families of the MAPK cascade were identified and reported from five different agriculturally and economically important crop species of the *Solanaceae* and *Rubiaceae* families based on conserved signature motifs aligned throughout the members of the families under this gene superfamily. Genes reported from the species after strict filtering were: 89, tomato; 108, potato; 63, eggplant; 79, pepper; 64, coffee. These MAPKs were found to be randomly distributed throughout the genome on the chromosomes of the respective species. Various characteristics of the identified genes were studied including gene structure, gene and coding sequence length, protein length, isoelectric point, molecular weight, and subcellular localization. Moreover, maximum likelihood test of phylogeny was conducted on the retrieved sequences for the three MAPK cascade families to determine their homologous relationships which were also analyzed quantitatively by heat plots.

## Introduction

Mitogen-activated protein kinases (MAPKs) are a crucial superfamily of genes involved in plant processes, from the very basic to the essentially complex. MAPK cascade members are known to be involved in basic cell processes including cell division, development, and signaling (Qiu et al., 2008; Teige et al., 2004; Zhang & Klessig, 2001). As part of the MAPK cascade, these genes work in relation to each other, by working upstream and downstream to certain members by phosphorylation at defined motif sites (Ichimura et al., 2002). The activation of these genes depend upon the phosphorylation events occurring at various levels of the MAPK cascade; mitogen-activated protein kinase kinase kinases (MAPKKKs, MKKK) being at the top level are phosphorylated as a result of external stress or internal growth signals, in turn activating the mitogen-activated protein kinase kinases (MAPKKs, MKK) which act on mitogen-activated protein kinases (MAPKs, MPK) which, being at the lowest level of the cascade, act on the final substrates (Khokhlatchev et al., 1998). Members of these families have been known to show functional redundancy with their paralogs (Asai et al., 2002; Krysan et al., 2002; Rodriguez, Petersen & Mundy, 2010).

Arabidopsis, being a model plant due to its ideal genomic characteristics and growth, used widely as a reference in Plant Science research, has been identified with 80 members in the MAPKKKs, including 21 MEKK-like, 48 Raf-like, and 11 ZIK genes, 10 members in the MKKs, and 20 members in the MPKs (Ichimura et al., 2002). Members of the MAPK cascade have been identified and reported in various plant species during recent times including rice (Rao et al., 2010), maize (Kong et al., 2013), cotton (Yin et al., 2013) and canola (Liang et al., 2013).

Angiosperms are known to quite commonly undergo polyploidization (Masterson, 1994; Stebbins Jr, 1950) and diploidization again with the passage of time due to genetic loss (Wolfe, 2001). Triplications also seem to have happened in tomato and potato leaving few genes triplicated (Consortium, 2012); however, polyploidy has not been found in a group of extant cultivated species from *Solanaceae*. This group includes tomato, potato, eggplant, and pepper, with evolutionarily conserved karyotype mostly with *x* = 12 basal number of chromosomes (Olmstead et al., 2008; Robertson, Goldberg & Igic, 2011). *Solanaceae* is a large and diverse family of Angiosperms in the plant kingdom, based on herbs, shrubs, short trees, and important crops like tomato, potato, eggplant, and pepper with ≈100 genera consisting of 2.5 k species (Knapp et al., 2004; Olmstead & Bohs, 2006).

*Coffea canephora*, one of the two parents contributing its genome to the formation of common coffee, *Coffea arabica*, is a member of the family *Rubiaceae*, one of the largest families among Angiosperms based on 660 genera containing ≈11 k species (Lashermes et al., 1999; Robbrecht & Manen, 2006). Coffee is used worldwide and has a high production and consumption rate. It was selected as one of the subject crop species in this study due to the fact that its genome is closely related to the species belonging to the family *Solanaceae*, and the architecture of their chromosomes is also quite similar (Lin et al., 2005).

The rapid availability of whole genome sequences in plant genome databases calls for a need for identifying and characterizing the fundamental gene families conserved throughout the plant kingdom, few of which being the families and subfamilies of the MAPK cascade. Here we report MPKs, MKKs, and MAPKKKs from the recently sequenced genomes of tomato, potato, eggplant, pepper, and coffee, all of which are cultivated and used as crops worldwide. The fact that they have a worldwide usage raises the importance of identification and characterization of plant genes involved in plant defense and stress responsiveness in these crops. Gene identification based on conserved regions was followed by a detailed study of gene characteristics; most importantly, gene positions were recorded against chromosomes of the crop genomes to get an insight into their distribution over the chromosomes. The identified sequences were also used to construct phylogenetic trees and correlation plots for the families and subfamilies to study the relationship among themselves and with the reference set of genes from Arabidopsis.

## Methods

### Database mining

CDS and protein sequences from the genomes of tomato, potato, eggplant, pepper, and coffee were extracted from the Solgenomics network (Bombarely et al., 2011) *via* BLAST, based on a profile generated with high sensitivity for *e*-value (threshold: 1e−10) and overall scores for identity (>70%) and similarity (>50%), using our reference dataset based on AtMPK, AtMKK, and AtMAPKKK sequences retrieved from TAIR (Lamesch et al., 2012). In all, 20 MPK, 10 MKK, and 80 MAPKKK, including 21 MEKK-like, 11 ZIK, and 48 Raf-like protein sequences from Arabidopsis were retrieved and used as a reference dataset. Table 1 shows the details of the plants in this study and the genome databases utilized for their sequence retrieval.

**Table 1:** Scientific names of the subject crop plants and their database versions employed in retrieving sequences.

Species	Scientific name	Abbreviation	Database
Tomato	*Solanum lycopersicum*	Sl	Tomato Genome CDS (ITAG release 2.40)
			Tomato Genome protein sequences (ITAG release 2.40)
Potato	*Solanum tuberosum*	St	Potato PGSC DM v3.4 CDS sequences
			Potato PGSC DM v3.4 protein sequences
Eggplant	*Solanum melongena*	Sm	Eggplant Genome CDS (release 2.5.1)
			Eggplant Genome protein sequences (release 2.5.1)
Pepper	*Capsicum annuum*	Ca	*Capsicum annuum* cv CM334 Genome CDS (release 1.55)
			*Capsicum annuum* cv CM334 Genome protein sequences (release 1.55)
Coffee	*Coffea canephora*	Cc	*Coffea canephora* CDS v1.0
			*Coffea canephora* protein sequences v1.0

### Identification and sequence analysis

The initial pool of data was tested for the conserved domains and signature motifs of the subfamilies, and the sequences lacking those conserved regions were eliminated from the dataset. The analysis of conserved regions was performed using PROSITE (release 20.109) at the ExPASy server. Sequences in the hence formed dataset were subjected to multiple sequence alignments using Geneious 9.1.5 (Kearse et al., 2012) and conservation of motif positions among the sequences was observed. Distance matrices of these sequences were also generated by Geneious in order to have a quantitative insight into the pairwise sequence similarities among the homologs for all the subject families and subfamilies.

### Phylogenetic tree construction

Phylogenetic trees of the selected sequences were constructed using the Maximum Likelihood (ML) method for statistical calculation and a phylogeny test was conducted with 1,000 replications, *via* bootstrapping in MEGA6.0 (Tamura et al., 2013). This was done after alignment by the integrated ClustalW tool using these parameters: gap opening and extension penalty of 10 and 0.1 respectively for pairwise alignment, 10 and 0.2 respectively for multiple alignment; BLOSUM protein weight matrix based on residue-specific and hydrophilic penalties, and a gap separation distance of 4. The substitution model employed for phylogeny reconstruction was the Jones-Taylor-Thornton (JTT) model with uniform rates among sites and partial deletion with a 95% site coverage cutoff. The initial tree for ML was generated using Neighbor Joining (NJ/BioNJ) and the ML heuristic method used was Nearest-Neighbor-Interchange (NNI) with a very strong branch swap filter. These ML trees were submitted to the database of phylogenetic trees, TreeBASE (Wang et al., 2005).

Quantitative similarities among the CDS sequences of the genes identified in each gene family and subfamily along with the CDS of the reference genes were calculated in the form of percentages *via* Gegenees 1.0.4 (Agren et al., 2012) on a 64-bit windows platform after successfully importing these CDS sequences to a locally generated database in Gegenees. These values were used by the tool to generate correlation plots for each family separately by alignment *via* BlastN using the default parameters. This data was then exported to SplitsTree4.14.3 (Huson & Bryant, 2006) in the form of a distance matrix in order to construct a distance-based UPGMA tree. The correlation plot was stored after manual sorting according to the UPGMA tree with the default color profile 1:4.

### Gene structure and distribution of identified genes on chromosomes of respective crops

Identified genes from all MAPK cascade families belonging to tomato, potato, pepper, and coffee were studied for exon-intron structure using JBrowse (Skinner & Holmes, 2010) and those belonging to eggplant by GSDS 2.0 (Hu et al., 2015). The number of exons in each one of them was recorded in the respective Tables S1–S5. Various characteristics of the genes were studied, including gene, CDS, and protein size (Tables S1–S5). Molecular weight and isoelectric point (pI) were predicted using the Compute pI/Mw tool (Bjellqvist et al., 1993) while localization of the genes within the cell based on their properties was predicted *via* CELLO v.2.5 (Yu et al., 2006) as shown in Tables S1–S5.

Genes from the selected species were subjected to BlastN against whole genome to elucidate their positions on chromosomes of the respective species. Gene positions in tomato were elucidated according to SL2.40 WGS chromosomes while those in potato from ST3.10 WGS chromosomes. In case of pepper genes, positions were predicted from whole genome chromosomes version 1.55 while for coffee genes, positions were based on genome version 1.0. All 89 genes of MAPK cascade families in tomato and 108 genes in potato were successfully mapped to their respective 12 chromosomes. Out of the total 79 genes of MAPK cascade families in pepper, 74 were successfully mapped to 12 chromosomes; however, five were present in uncharacterized chromosomal regions. A total of 60 of the 64 CcMAPKs mapped to 11 coffee chromosomes while four of these genes were in uncharacterized regions.

## Results and Discussion

### Sequence extraction, identification and analysis

A significant number of members in the three families of the MAPK cascade were reported from five important crop species of the *Solanaceae* and *Rubiaceae* families, including tomato, potato, eggplant, pepper, and coffee, all of which are used as food worldwide. Identification of the three complete families of the MAPK cascade has not been done earlier in potato, eggplant, pepper, and coffee. However, being widely studied due to their vital functions, a few of these genes have recently been identified and reported as MPK, MKK, and MAPKKK in tomato (Daigle & Matton, 2015; Kong et al., 2012; Lazar et al., 2014; Mohanta et al., 2015; Wu et al., 2014); as MPK, MKK, and MEKK-like from MAPKKK in potato (Daigle & Matton, 2015; Lazar et al., 2014; Mohanta et al., 2015); and, as MPK and MKK in pepper (Liu et al., 2015). However, genome identification of the three complete gene families hasn’t been reported for any one of them; thus, such an identification will be a huge step forward towards unraveling their functionality and importance. In this study, we identified genes of MPKs, MKKs, and MAPKKKs (including MEKK-like, Raf-like and ZIK) from tomato, potato, eggplant, pepper, and coffee and compared them with a few of the genes reported in recent literature belonging to these families. Sequences for the three families of MAPK cascade were extracted from the genomes of interest based on overall score and e-value threshold of 1e−10; 121 sequences from the tomato genome protein sequences, and 159, 130, 124, and 87 from potato, eggplant, pepper, and coffee, respectively. Sequence duplications (100% identity) were removed from the dataset.

The extracted sequences in this initial dataset were subjected to domain and motif scanning for generating a refined dataset based on conservation of signature motifs in each subfamily and absence of such motifs was used as a criterion for elimination of sequences from the dataset. The total number of sequences in each family and subfamily retrieved from the genomes as a final dataset are represented in Table 2. The accession numbers of these sequences from the subject plants have been put together in Table S6. All these families had a substantial increase in variety in the potato genome over the course of evolution. The extremely large sizes of the genomes of subject species in comparison to the small genome size of Arabidopsis does not have much effect on the sizes of the gene families in the MAPK cascade in these species. Genes in the MAPK cascade are involved in a variety of signaling mechanisms even though MAPKKKs are very large in number compared to MPKs and MKKs.

**Table 2:** Total number of sequences as in the final dataset of the subject plant species.

Plant	Total no. of sequences	MAPK superfamily				
		MPK	MKK	MAPKKK		
				MEKK-like	Raf-like	ZIK
Sl	89	14	4	17	44	10
St	108	21	6	22	43	16
Sm	63	12	4	14	28	5
Ca	79	14	5	17	37	6
Cc	64	12	4	12	28	8

Collected sequences in various datasets belonging to MPK, MKK, and MAPKKK gene families of the selected plant species were subjected to multiple sequence alignments and searched for the signature motifs of each defined family. The gene family-wise multiple sequence alignments of the sequences in the datasets, shown in Figs. S1–S5, depict the perfect conservation of the signature motifs throughout the datasets, which contain various numbers of sequences in the respective families from these plants. This presence of perfectly aligned motifs in the homologs is the characteristic that denotes the members of these families and subfamilies and distinguishes them from proteins of other families. This conservation data also suggests that the function in these genes has been retained throughout the evolution of these species since the active site bearing motifs remain intact.

Distance matrices were generated using the sequence data from each family and subfamily to analyze the percentage similarity among the homologous protein sequences; these were used to study the quantitative aspect of homology. It was observed that the similarity among the homologous sequences was higher for MPK and MKK families in contrast to the greater divergence seen in the MAPKKK family (Figs. S6–S10). Alongside the conserved regions that assure gene functionality, the high rate of conservation along the whole sequence length among some of the paralogs accounts for functional redundancy.

### Phylogenetic analysis

Phylogenetic trees from the dataset reveal the clustering pattern of the protein sequences with those in the reference dataset of Arabidopsis. Sequences were observed in various categories based on identity. Sequences in various groups of the MAPK cascade families were seen to cluster with the respective members in the reference dataset. A total of 89 sequences from tomato were used in the phylogenetic analysis, most of which have also been reported very recently (Wu et al., 2014). Furthermore, 108 sequences from potato, 63 from eggplant, 79 from pepper, and 64 from coffee have been reported in this study based on perfectly conserved signature motifs belonging to the three major families of the MAPK cascade. Although, few members of some of these families were reported recently (Daigle & Matton, 2015; Kong et al., 2012; Lazar et al., 2014; Liu et al., 2015; Mohanta et al., 2015; Wu et al., 2014), this articles reports the largest set of genes identified from all three of these gene families and their further subfamilies in the said species. Phylogenetic analysis of the reported sequences shows the conservation of the genes throughout the *Solanaceae* plant family and coffee, which is closely related to *Solanaceae* species in terms of its genetic make-up. Also, the order of phylogeny of the studied species was seen to be maintained. The ML trees can be viewed and accessed interactively at TreeBASE: http://purl.org/phylo/treebase/phylows/study/TB2:S19994.

#### Mitogen-activated protein kinases

**Figure 1:**
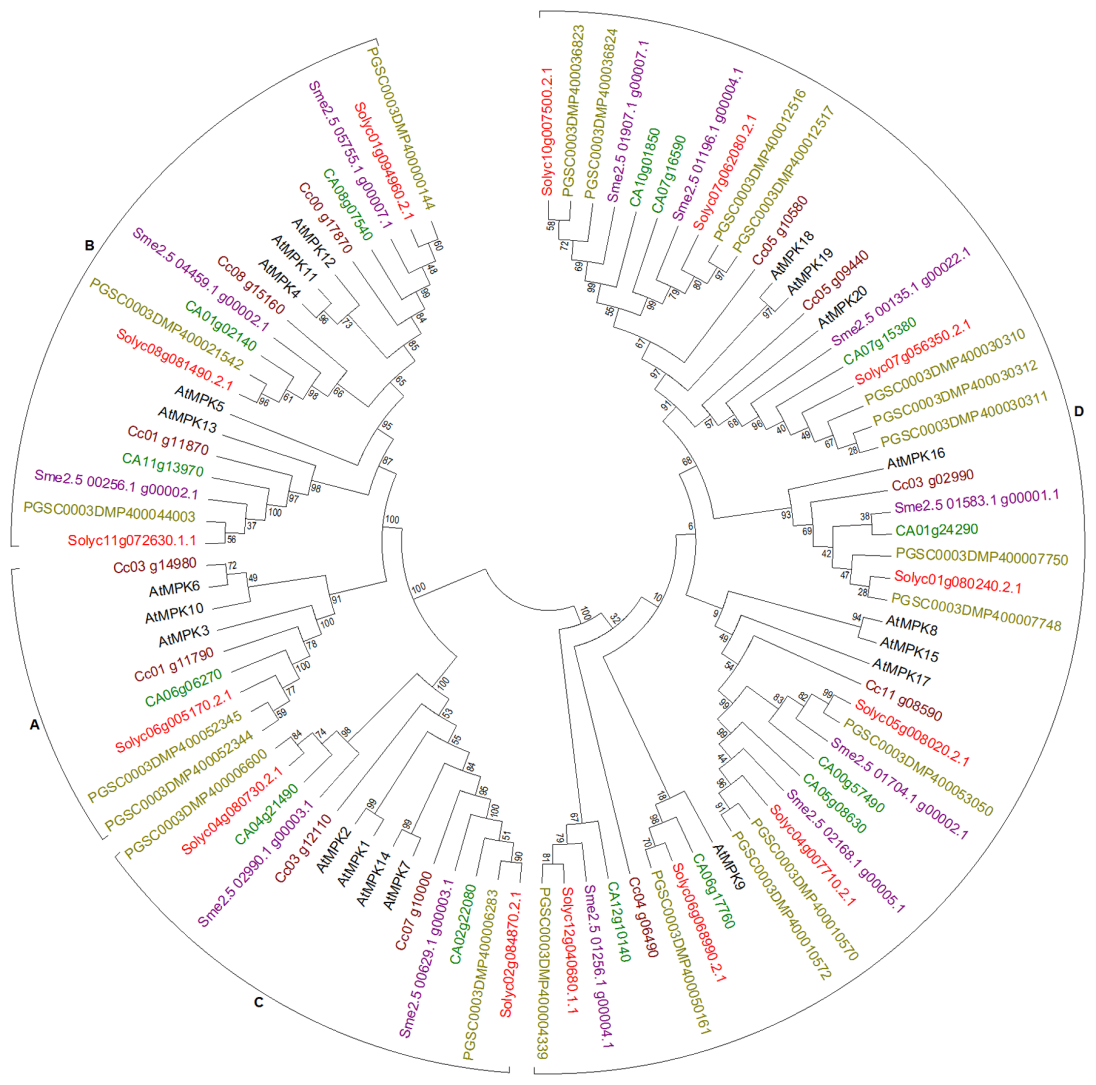
Phylogenetic tree of MPK genes from tomato, potato, eggplant, pepper, and coffee in reference to Arabidopsis clustered according to their phylogeny. Tree generated by maximum likelihood method using 1,000 bootstrapping replicates. Color code: tomato, red; potato, olive; eggplant, purple; pepper, green; coffee, maroon; Arabidopsis, black.

Sequences with perfectly conserved signature motifs were used for generation of the final phylogenetic trees. In MPK family of the MAPK cascade, 14 sequences with complete conserved signature motif as in the reference dataset of Arabidopsis were extracted from tomato, 21 from potato, 12 from eggplant, 14 from pepper, and 12 from coffee genome. In MPK family, the genes were found to cluster with reference AtMPK sequences forming respective clades, A, B, C, and D (Fig. 1), as previously known for Arabidopsis (Ichimura et al., 2002). The accession numbers of the genes from various plants have been colored in the figure for ease of reading. Two sequences each from potato and coffee, while one sequence each from tomato and pepper were seen to cluster in group A. Three sequences from each of the five subject species clustered with Arabidopsis group B genes, while two from each of them clustered with group C genes. Group D cluster had rather variable number of MPK genes from these species. With eight genes each from tomato and pepper, 14 from potato, seven from eggplant, and five from coffee, this cluster was the largest in the MPK tree.

Additionally, sequences with signature motifs clustering at appropriate clades in the phylogenetic trees but with minor substitution (changes in 1–3 amino acids) in the conserved region of the motif were eliminated from the final trees shown in Fig. 1 and the following figures. Since signature motifs define the family and the conserved amino acids in this region are responsible to perform certain functions, changes in these amino acids lead to loss of functionality and hence the protein no longer holds that identity if the changes are permanent over the course of evolution. Thus these sequences were removed from the dataset of MAPK cascade members. For MPKs, these include three sequences from tomato, one from potato, six from eggplant, and three from pepper (See Table S7 for information of accession numbers of these sequences). Three incomplete sequences but with signature motif were eliminated from eggplant dataset (See Table S7 for accession numbers).

#### Mitogen-aactivated protein kinase kinases

**Figure 2:**
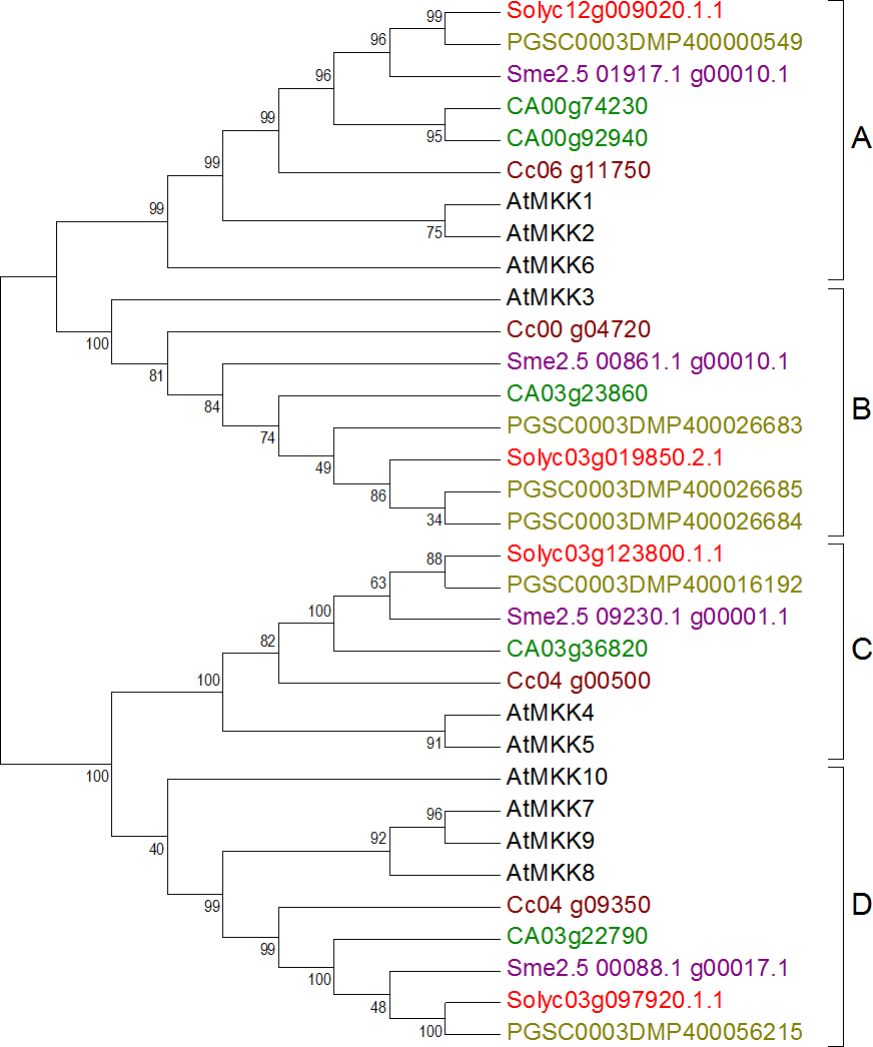
Phylogenetic tree of MKK genes from tomato, potato, eggplant, pepper, and coffee in reference to Arabidopsis clustered according to their phylogeny. Tree generated by maximum likelihood method using 1,000 bootstrapping replicates. Color code: tomato, red; potato, olive; eggplant, purple; pepper, green; coffee, maroon; Arabidopsis, black.

Sequences with less conserved motif, eliminated from the dataset, include one each from tomato, potato, and pepper, three from eggplant, and two from coffee (See Table S7 for information of accession numbers of these sequences). The final dataset of the MKK family was composed of four sequences from tomato, six from potato, four from eggplant, five from pepper, and four from coffee in reference to the 10 reference sequences from Arabidopsis. The phylogenetic tree constructed from this dataset showed four sub-groups A, B, C, and D with significant bootstrap values and in accordance with the literature (Hamel et al., 2006). Two sequences from pepper and one each from the other plants fall in group A, three sequences from potato and one each from rest of the plants fall in group B, while one sequence from each plant was categorized in each of the two remaining groups C and D as shown in Fig. 2. Clustering is as previously known in Arabidopsis (Ichimura et al., 2002).

#### Mitogen-activated protein kinase kinase kinases

Sequences collected from the genomes for the largest family of the MAPK cascade were further analyzed by breaking them down into three datasets according to the three subfamilies it is composed of, with individually defined signature motifs, for the ease of data handling and output view.

Sequences extracted from tomato for MKK and MAPKKK were in accordance with the recent study (Wu et al., 2014), along with 9 more sequences (Solgenomics accession numbers: Solyc02g085620.2.1, Solyc04g071120.2.1, Solyc02g083290.2.1, Solyc08g065250.2.1, Solyc09g082470.2.1, Solyc03g114210.2.1, Solyc05g013070.2.1, Solyc09g091460.2.1, Solyc02g031910.2.1) in Raf-like subfamily.

**Figure 3:**
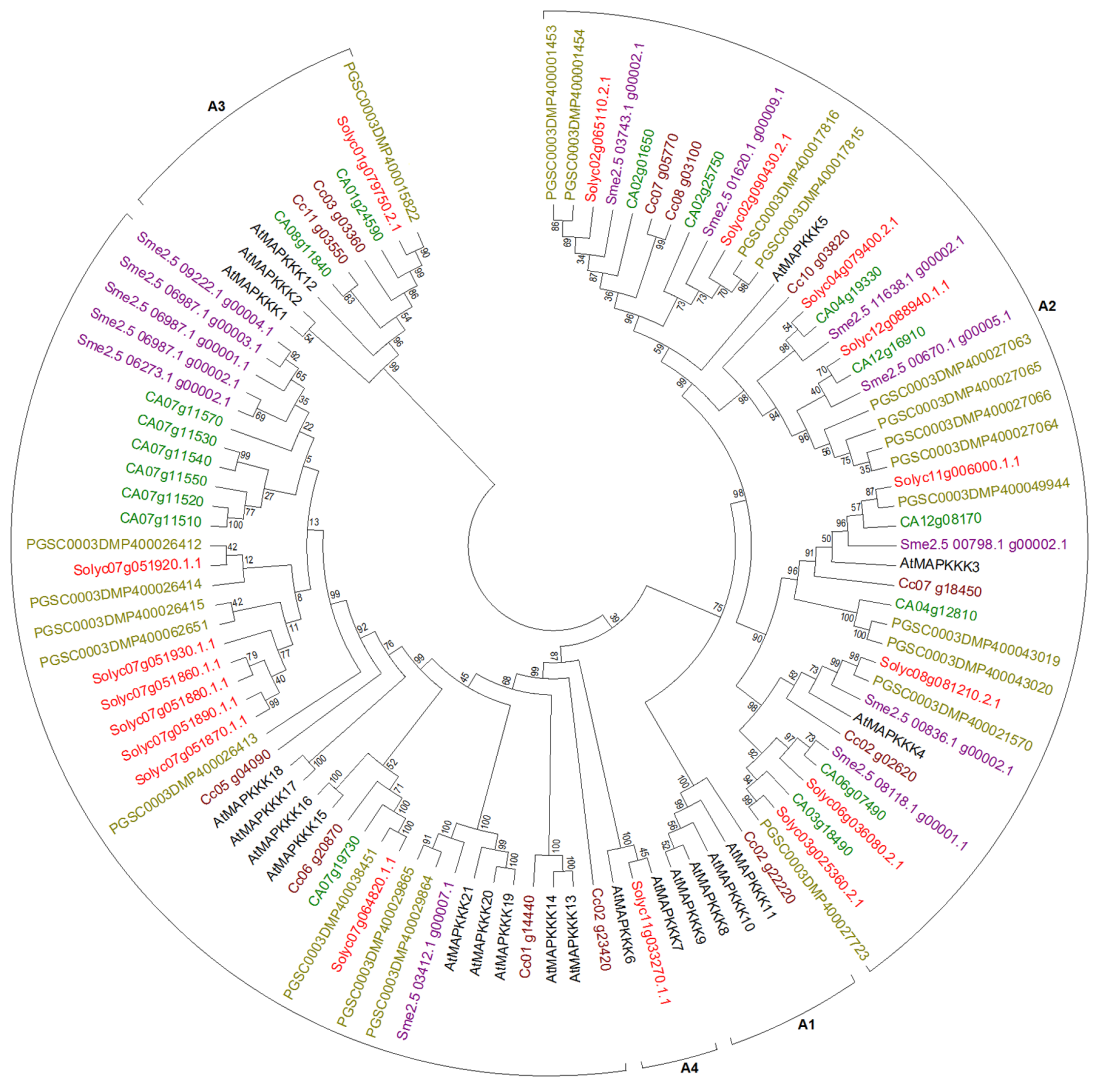
Phylogenetic tree of MEKK-like genes from tomato, potato, eggplant, pepper, and coffee in reference to Arabidopsis clustered according to their phylogeny. Tree generated by maximum likelihood method using 1,000 bootstrapping replicates. Color code: tomato, red; potato, olive; eggplant, purple; pepper, green; coffee, maroon; Arabidopsis, black.

Partial sequences containing MEKK-like signature motif were removed from the final dataset used for tree generation, including two sequences from eggplant and one from coffee (See Table S7 for accession numbers). Six sequences from tomato and potato each, 11 from eggplant, seven from pepper, and one from coffee (See Table S7 for information of accession numbers of these sequences) were removed from the final dataset due to incomplete conservation in the motif region. Phylogenetic tree for MEKK-like genes from the plants was generated using the final dataset (Fig. 3). This tree showed 1 coffee gene clustered to the Arabidopsis genes in clade A1, and one tomato gene in clade A4. Clade A2 showed clustering of eight tomato, 13 potato, seven eggplant, eight pepper, and five coffee genes with their orthologs in Arabidopsis. One gene each from tomato and potato along with two genes each from pepper and coffee clustered in the clade A3. The fifth clade in this tree was the largest with seven genes each from tomato and pepper, eight from potato, six from eggplant, and four from coffee, clustering to Arabidopsis orthologs. Multiple duplications were seen in tomato, potato, eggplant, and pepper as shown by the multiple number of their MEKK-like genes clustered together in this clade. Clustering is according to literature (Ichimura et al., 2002).

**Figure 4:**
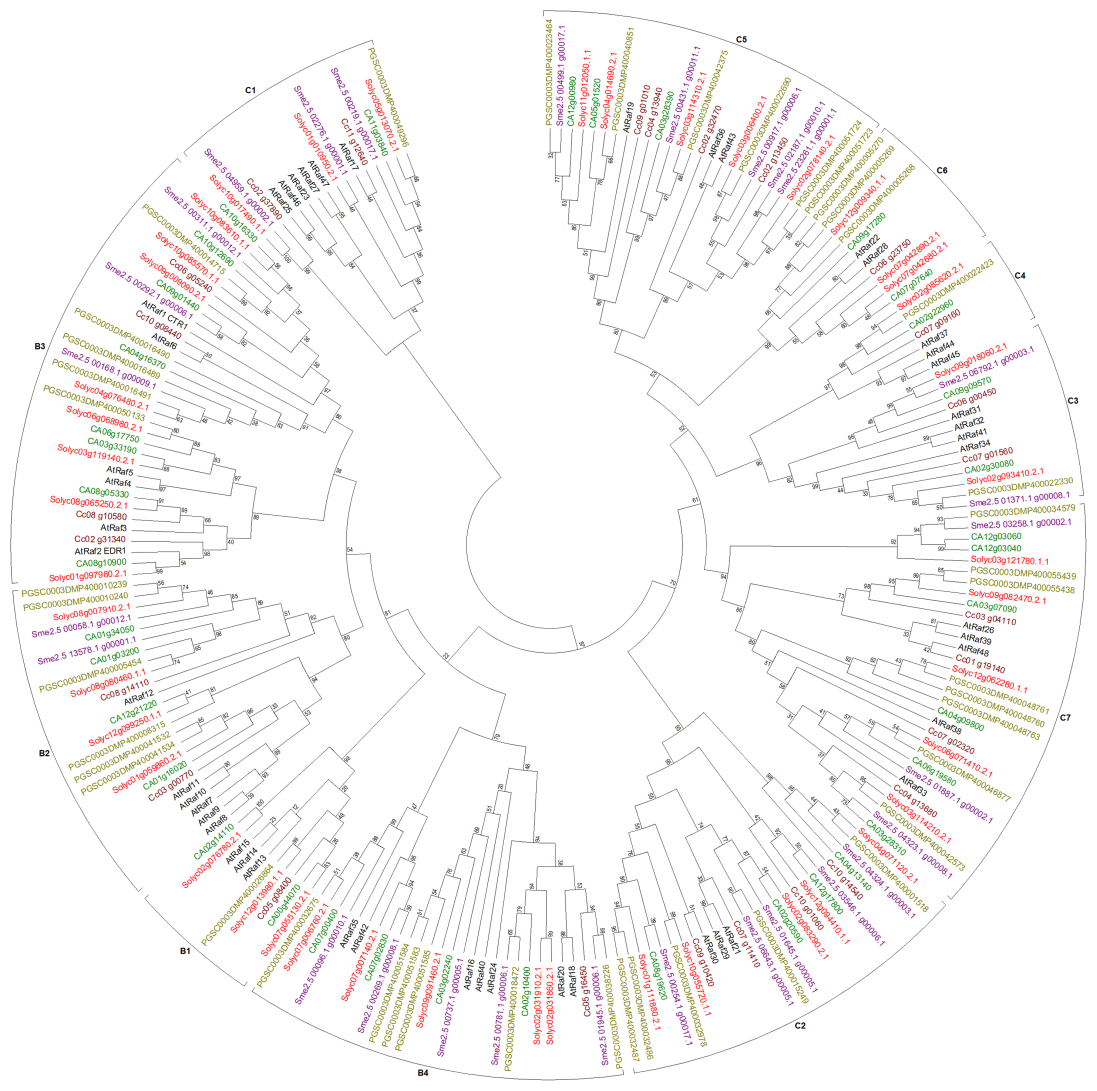
Phylogenetic tree of Raf-like genes from tomato, potato, eggplant, pepper, and coffee in reference to Arabidopsis clustered according to their phylogeny. Tree generated by maximum likelihood method using 1,000 bootstrapping replicates. Color code: tomato, red; potato, olive; eggplant, purple; pepper, green; coffee, maroon; Arabidopsis, black.

Due to minor changes in the motif region of the amino acids, large number of sequences were removed from the final dataset of Raf-like proteins, including 14 sequences from potato, six from eggplant, five from pepper, and one from coffee (See Table S7 for information of accession numbers of these sequences). Based on partiality of the sequences, two were removed from eggplant dataset, five from pepper, and four from coffee (See Table S7 for accession numbers). Figure 4 represents the final phylogenetic tree of the Raf-like proteins from our plants. B1 cluster comprised of three genes from tomato, one each from potato and coffee, and two genes from pepper clustered to three Arabidopsis genes. Four each tomato and pepper genes, six potato genes, two each eggplant and coffee genes clustered in group B2. Clade B3 had rather variable number of genes with eight tomato and pepper each, five potato, three eggplant, and four coffee genes clustering to Arabidopsis orthologs. Five genes each from tomato and eggplant, six potato, four pepper, and one from coffee made the B4 clade by clustering with seven Arabidopsis genes. In the clade C1, there were three genes each from tomato and eggplant, one each from potato and pepper, and two from coffee. Similarly, clade C2 comprised of five genes each from tomato, potato, and eggplant, while four each from pepper and coffee. Clade C3 was formed of two genes from each of the subject species except potato which had only one gene clustering to the four Arabidopsis genes in this clade. Only single genes from each of the subject species except eggplant made up the shortest clade C4 in this tree. Cluster C5 comprised of five genes each from tomato and eggplant, six from potato, three from pepper, and four from coffee clustered to three orthologs from Arabidopsis. Three sequences each from tomato and potato, two from pepper, and a single one from coffee clustered with two Arabidopsis genes to form the C6 clade. The last clade of Raf-like tree, C7, had five tomato, eight potato, three eggplant, six pepper, and four coffee genes clustered with Arabidopsis genes. Clustering of the sequences with Arabidopsis were in accordance with the reported nomenclature (Ichimura et al., 2002).

**Figure 5:**
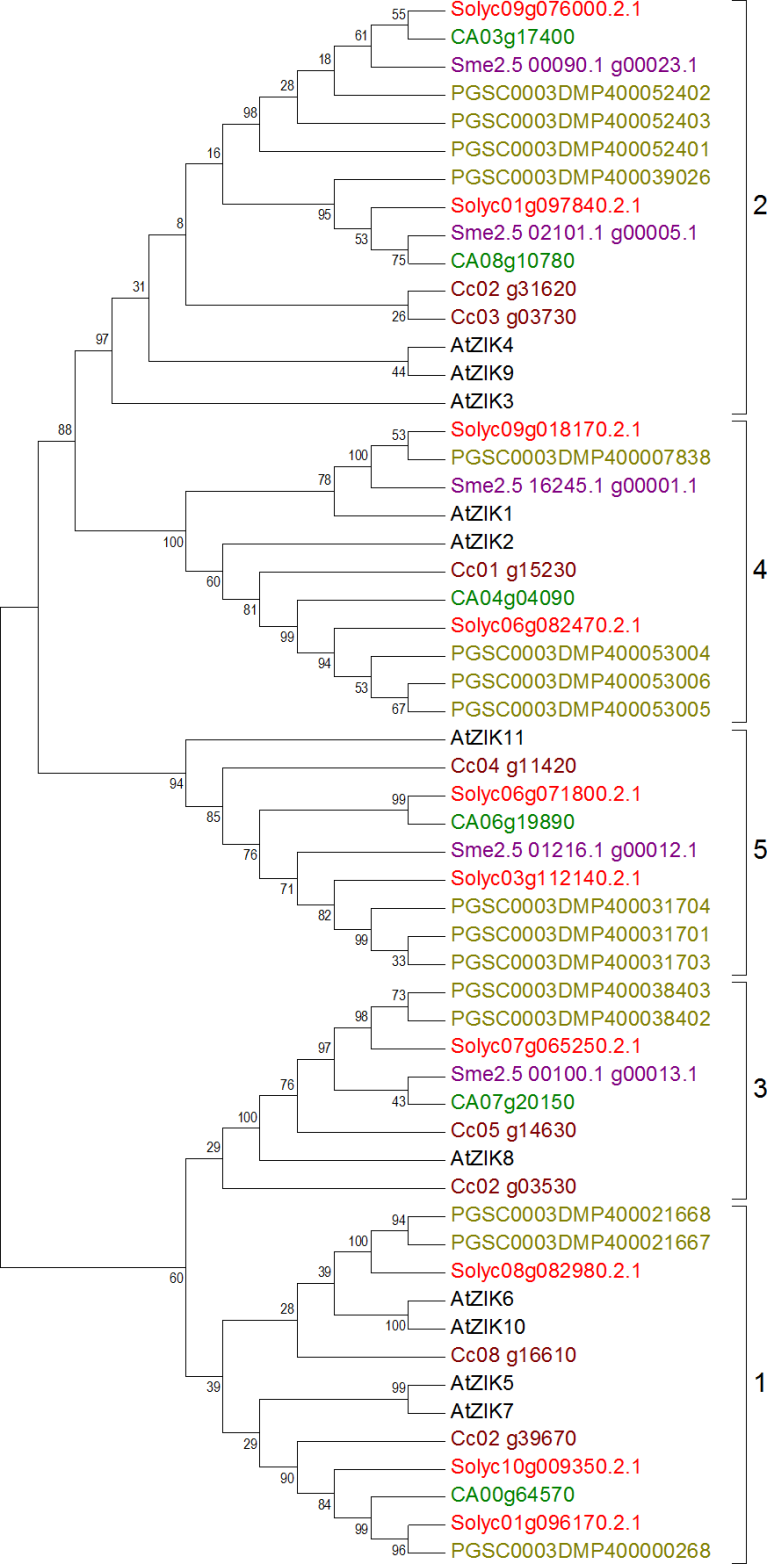
Phylogenetic tree of ZIK genes from tomato, potato, eggplant, pepper, and coffee in reference to Arabidopsis clustered according to their phylogeny. Tree generated by maximum likelihood method using 1,000 bootstrapping replicates. Color code: tomato, red; potato, olive; eggplant, purple; pepper, green; coffee, maroon; Arabidopsis, black.

ZIK genes seem to have a sister clade with Raf-like genes but due to huge number of sequences in the datasets, ZIK was analyzed separately for phylogeny. Similar to the previous subfamilies, sequences containing the signature motif with small alterations in the conserved motif region were removed from the final dataset of ZIK; these include four sequences from tomato, two from eggplant, three from pepper, and one from coffee (See Table S7 for information of accession numbers of these sequences). One incomplete sequence was also removed from the eggplant dataset (See Table S7 for accession number). The tree constructed with the final dataset was observed to have properly clustered sequences from all species (Fig. 5). The first cluster of the ZIK tree formed as a result of clustering of 13 genes: three each from tomato and potato, two from coffee, one from pepper, and four from Arabidopsis. The second cluster constituted of 15 genes: three from Arabidopsis, four from potato, and two each from the rest of the species. The third cluster was shorter with two sequences each from potato and coffee, and a single gene from each of the remaining species. Likewise, the fourth cluster had four genes from potato, two each from tomato and Arabidopsis, and one each from the other species. Lastly, the fifth cluster comprised of three genes from potato, two from tomato, and one each from all other species.

Thorough phylogenetic analysis of the MAPK cascade families in these related plant species revealed various number of members compared to the reference model plant, Arabidopsis. The genes reported here have been numbered (Gene names in Tables S1–S5) in accordance to the nearest clustered Arabidopsis homolog in the phylogenetic tree. The slight differences in number of genes belonging to each family and subfamily of the MAPK cascade in each species reported here in contrast to those reported earlier is due to strict filtering criteria used here based on exclusion of the sequence from the dataset even in case of few mutations in the motif region. Hence, few of the sequences being reported previously as being members of these families were discarded from the dataset presented here and have been mentioned in the subsections of this section.

### Quantitative phylogenetic analysis

The analysis for assessing the range of similarity, shared among the genes of each family and subfamily, was performed by an all versus all similarity search at the gene level. Taking into account all the identified as well as reference gene sequences, a correlation plot was generated and analyzed for each family and subfamily separately. Variable amount of similarity was seen among the genes. The correlation plot and the distance-based phylogenetic tree, both generated on the basis of content sharing, were then analyzed (Figs. S11–S15).

At the genetic level, AtMPK1 formed a new branch, while rest of the genes from all the species were seen to make clusters together, which means that AtMPK1 among the MPK genes had the least similarity with genes in this family irrespective of being a part of any subject species genome. Similarly, similarities shared among the genes can be seen in the five correlation plots generated for the genes of each family and subfamily (Figs. S11–S15). Quantitative similarity measure among the genes of each gene family *via* distance matrix as well as correlation plot analysis according to phylogenetic tree showed variable similarity indices among the orthologs as well as the paralogs.

### Structural organization of genes

Gene structures of genes identified from tomato, potato, eggplant, pepper, and coffee were studied. Members belonging to group A, B, and C of MPK had uniformity in gene structure within the groups while members belonging to the group D MPKs were variable in number, characteristics and length (Fig. S16, Table S8). Although similar in gene structures, their lengths were found to be variable. Thus, similarity was seen among genes from all the species with respect to the clade they form in the tree according to the group to which they belong. Genes in group A, B, and C had the highest similarity in gene structure among the respective group members.

Similarly, members identified as MKK had similarity in gene structures but differences in gene lengths (Fig. S17, Table S8). In case of MKK genes, group A members had similarities among themselves, and so did the respective members of group C and D, while group B members did not show identity in gene structure among themselves.

The gene structures of MEKK-like genes identified from the crops’ genomes are shown in Fig. S18. MEKK-like tree had five distinct clusters. Only one cluster, A4, in the tree had one very large gene from tomato with 23 introns. Contrary to the first cluster A2, which had the least gene structure similarities among the members, the rest of the clusters had gene structural similarities among their respective members that grouped together in the tree (Fig. S18, Table S8).

A total of 11 distinct clusters from Raf-like tree showed uniformity in some clusters and high variability in others (Fig. S19, Table S8). Except for the five clusters B1, B3, B4, C2, and C7, genes that clustered together in the rest of the groups had a uniform gene structure.

Five distinct clusters containing ZIK members displayed similar gene structure variability (Fig. S20, Table S8). ZIK genes had the highest variability in their gene structures even in the orthologs clustering together in the tree. Hence, gene structural uniformity was a characteristic of most of the genes grouping together in the tree except that this behavior wasn’t observed among the genes falling under the ZIK category.

The protein sequences from these genes have variable sizes in all subject species ranging from several hundred amino acids to over 1,000 amino acids. Variability in gene structure was also observed with some genes containing as many as 24 exons, and others as low as one. However, genes within the same clade have conserved gene structure, with the exception of ZIK genes. These conserved structural patterns in the clades are representative of their origin as a result of tandem or segmental duplications in ancestral species with a few of them containing small evolutionary differences. However, during the evolution of these genomes, the genes of these gene families seem to have preserved their structural organization in terms of exons and introns. Consistency in the exon-intron structure of homologous genes of the species clustering together in the tree and differences with those clustering in other subgroups represents the conservation of gene functions across various genomes as well as development of diverse functionality with the occurrence of a previous duplication, respectively. Even genes from coffee showed gene structural uniformities with orthologous members from *Solanaceae* species grouping together in a clade. Evolutionary gene structural conservation among the orthologous members of these gene families in plant genomes was thus seen over time; however, divergence was achieved by the paralogs since they aren’t all redundant, which might be the reason for functional divergence as reported for cucumber by Wang et al. (2015).

### Gene distribution

Gene positions of MPKs, MKKs, and MAPKKKs were retrieved and recorded on the chromosomes of tomato, potato, pepper, and coffee. Eggplant genome still needs to be processed further for whole genome alignment against a reference set of genome, like tomato, so that the gene positions on eggplant chromosomes can be evaluated. Genes were distributed on all chromosomes of the rest of the species.

**Figure 6:**
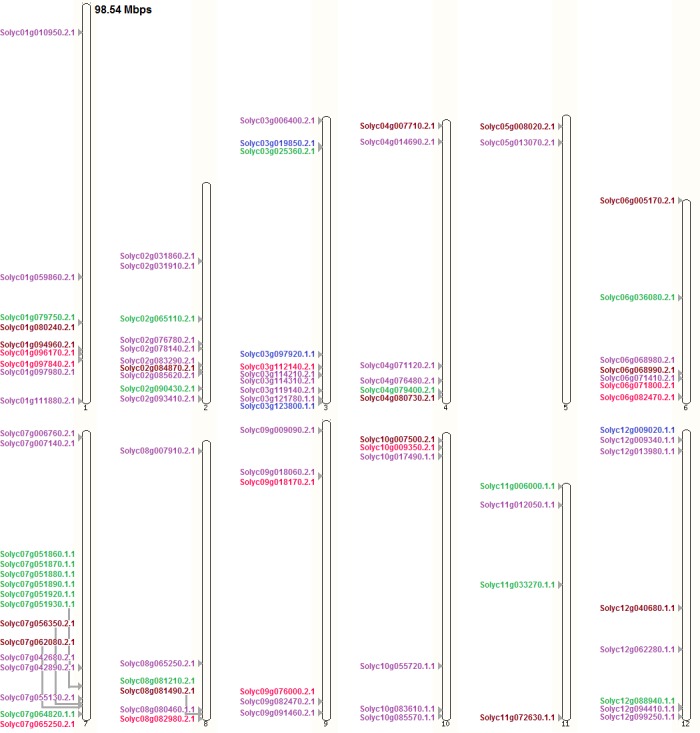
MPK, MKK, MEKK-like, Raf-like, and ZIK gene distribution on tomato chromosomes. Color code: maroon, MPK; blue, MKK; green, MEKK-like; purple, Raf-like; magenta, ZIK. Drawn to scale in reference to chromosome 1 labeled on top. Positions on the chromosomes ascending towards bottom.

Genes from all subfamilies were found to be distributed randomly over tomato chromosomes. MPK genes were found to be nearly equally distributed over the chromosomes. MKK genes were more concentrated on chromosome 3 along with a single member on top of chromosome 12, whereas chromosome 7 had the highest number of MEKK-like genes in tomato. Raf-like genes were wide-spread and present on every chromosome with the highest number of Raf-like genes on chromosome 2. The chromosomal gene distribution of all MAPK cascade subfamilies in tomato are shown in Fig. 6. According to Holub’s gene cluster (Holub, 2001), a single cluster was formed by 6 MEKK-like genes on chromosome 7 of tomato. However, if the test criteria weren’t kept strict to more than three genes to form a cluster, six clusters were formed on five of the 12 chromosomes by 16 of the genes reported, most of which were formed by two genes. These clusters were formed by the genes: Solyc01g097840.2.1 and Solyc01g097980.2.1 on chromosome 1 belonging to the ZIK and Raf-like subfamilies respectively; Solyc02g031860.2.1 and Solyc02g031910.2.1 on chromosome 2 both belonging to the Raf-like subfamily; Solyc03g114210.2.1 and Solyc03g114310.2.1 on chromosome 3 both belonging to the Raf-like subfamily; Solyc06g068980.2.1 and Solyc06g068990.2.1 on chromosome 6 belonging to the Raf-like subfamily and MPK family respectively; Solyc07g042680.2.1 and Solyc07g042890.2.1 on chromosome 7 both belonging to the Raf-like subfamily; Solyc07g051860.1.1, Solyc07g051870.1.1, Solyc07g051880.1.1, Solyc07g051890.1.1, Solyc07g051920.1.1 and Solyc07g051930.1.1 on chromosome 7 all belonging to the MEKK-like subfamily.

**Figure 7:**
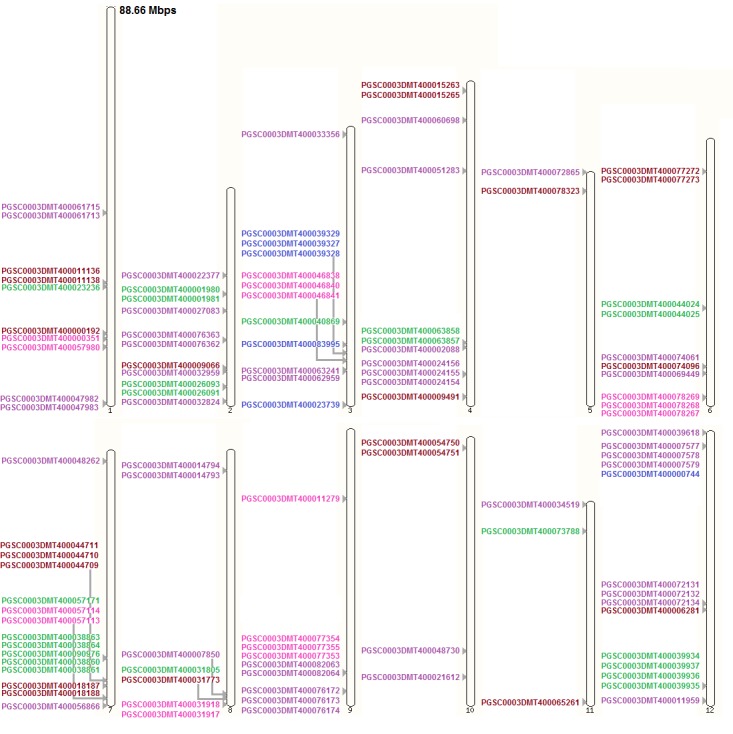
MPK, MKK, MEKK-like, Raf-like, and ZIK gene distribution on potato chromosomes. Color code: maroon, MPK; blue, MKK; green, MEKK-like; purple, Raf-like; magenta, ZIK. Drawn to scale in reference to chromosome 1 labeled on top. Positions on the chromosomes ascending towards the bottom.

Figure 7 shows the distribution of genes from MAPK cascade families and subfamilies on potato chromosomes. MPK genes in potato were found to be concentrated more on chromosomes 1, 4, and 6, while most of the MKK genes were found on chromosome 3 with one gene residing on chromosome 12. The genes of the largest MAPKKK subfamily, namely Raf-like, are distributed on all chromosomes with a dense concentration on chromosomes 2, 4, 9, and 12. Linked to the Raf-like subfamily is a small subfamily, ZIK, with the highest number of genes residing a single chromosome 9. Duplicated genes present on the same chromosomes were found to be closely distributed. According to the criteria for Holub’s gene clusters (Holub, 2001), five MEKK-like genes formed one cluster on chromosome 7 of potato. With a relaxed criteria, 47 of the genes reported here formed 19 clusters on seven of the 12 chromosomes of potato. Most of these clusters were formed by two genes. On chromosome 1, these include: PGSC0003DMT400061715 and PGSC0003DMT400061713 belonging to the Raf-like subfamily; PGSC0003DMT400011136 and PGSC0003DMT400011138 belonging to the MPK family; PGSC0003DMT400047982 and PGSC0003DMT400047983 belonging to the Raf-like subfamily. On chromosome 2, these include: PGSC0003DMT400001980 and PGSC0003DMT400001981 belonging to the MEKK-like subfamily; PGSC0003DMT400076363 and PGSC0003DMT400076362 belonging to the Raf-like subfamily; PGSC0003DMT400026093 and PGSC0003DMT400026091 belonging to the MEKK-like subfamily. On chromosome 3, these include: PGSC0003 DMT400063241 and PGSC0003DMT400062959 belonging to the Raf-like subfamily. On chromosome 4, these include: PGSC0003DMT400015263 and PGSC0003DMT400015265 belonging to the MPK family; PGSC0003DMT400063858 and PGSC0003DMT400063857 belonging to the MEKK-like subfamily. On chromosome 6, these include: PGSC0003DMT 400077272 and PGSC0003DMT400077273 belonging to the MPK family; PGSC0003DMT 400044024 and PGSC0003DMT400044025 belonging to the MEKK-like subfamily; PGSC0003DMT400074061 and PGSC0003DMT400074096 belonging to the Raf-like subfamily and MPK family respectively. Some of these clusters were formed by 3 genes each. On chromosome 3, these include: PGSC0003DMT400039329, PGSC0003DMT400039327 and PGSC0003DMT400039328 belonging to the MKK family; PGSC0003DMT400046838, PGSC0003DMT400046840 and PGSC0003DMT400046841 belonging to the ZIK subfamily. On chromosome 4, these include: PGSC0003DMT400024156, PGSC0003DMT400024155 and PGSC0003DMT400024154 belonging to the Raf-like subfamily. On chromosome 6, these include: PGSC0003DMT400078269, PGSC0003DMT400078268 and PGSC0003DMT 400078267 belonging to the ZIK subfamily. On chromosome 7, these include: PGSC0003DMT400044711, PGSC0003DMT400044710 and PGSC0003DMT400044709 belonging to the MPK family. On chromosome 12, these include: PGSC0003DMT400007577, PGSC0003DMT400007578 and PGSC0003DMT400007579 belonging to the Raf-like subfamily. Five genes on chromosome 7, PGSC0003DMT400038863, PGSC0003DMT400038864, PGSC0003DMT400090976, PGSC0003DMT400038860 and PGSC0003DMT400038861, all belonging to the MEKK-like subfamily formed a cluster.

**Figure 8:**
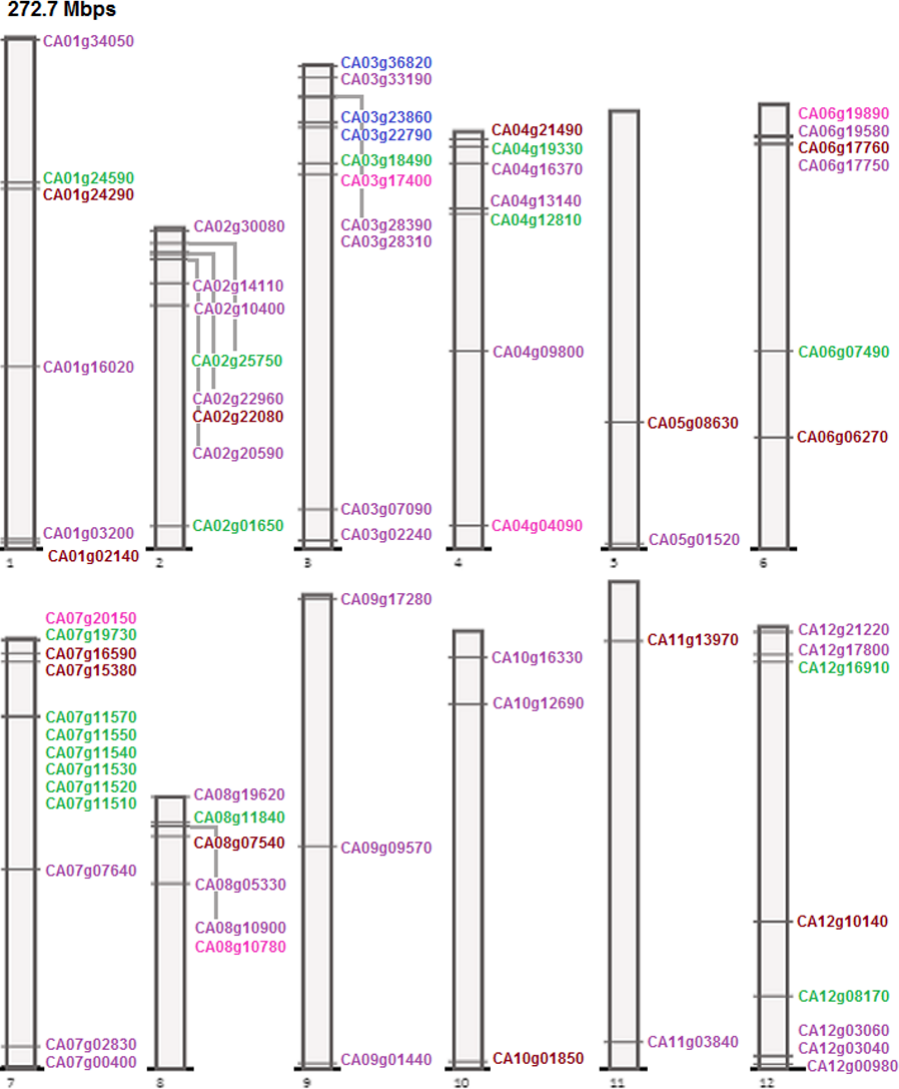
MPK, MKK, MEKK-like, Raf-like, and ZIK gene distribution on pepper chromosomes. Color code: maroon, MPK; blue, MKK; green, MEKK-like; purple, Raf-like; magenta, ZIK. Drawn to scale in reference to chromosome 1 labeled on top. Positions on the chromosomes ascending towards top.

A random distribution of genes belonging to these families and subfamilies was observed on pepper chromosomes with the highest concentration on chromosome 7. Pepper was identified with just three MKK genes all present on chromosome 3. Chromosome 7 had even the highest number of MEKK-like genes that are 7. Raf-like genes were distributed on all chromosomes with the highest population on chromosomes 2, 3, and 12 harboring five members each. Gene distribution on pepper chromosomes is shown in Fig. 8. As per definition of Holub’s gene cluster (Holub, 2001), one cluster was formed by six MEKK-like genes on chromosome 7 of pepper. However, with a relaxed criteria, 4 clusters were formed on 4 of the 12 chromosomes by 12 pepper genes. Three of these clusters were formed by two genes each: CA06g17750 and CA06g17760 on chromosome 6 belonging to the Raf-like subfamily and MPK family respectively; CA08g10780 and CA08g10900 on chromosome 8 belonging to the ZIK and Raf-like subfamilies; CA12g03040 and CA12g03060 on chromosome 12 both belonging to the Raf-like subfamily. One of the clusters was formed on chromosome 7 by 6 genes all belonging to the MEKK-like subfamily: CA07g11510, CA07g11520, CA07g11530, CA07g11540, CA07g11550 and CA07g11570.

**Figure 9:**
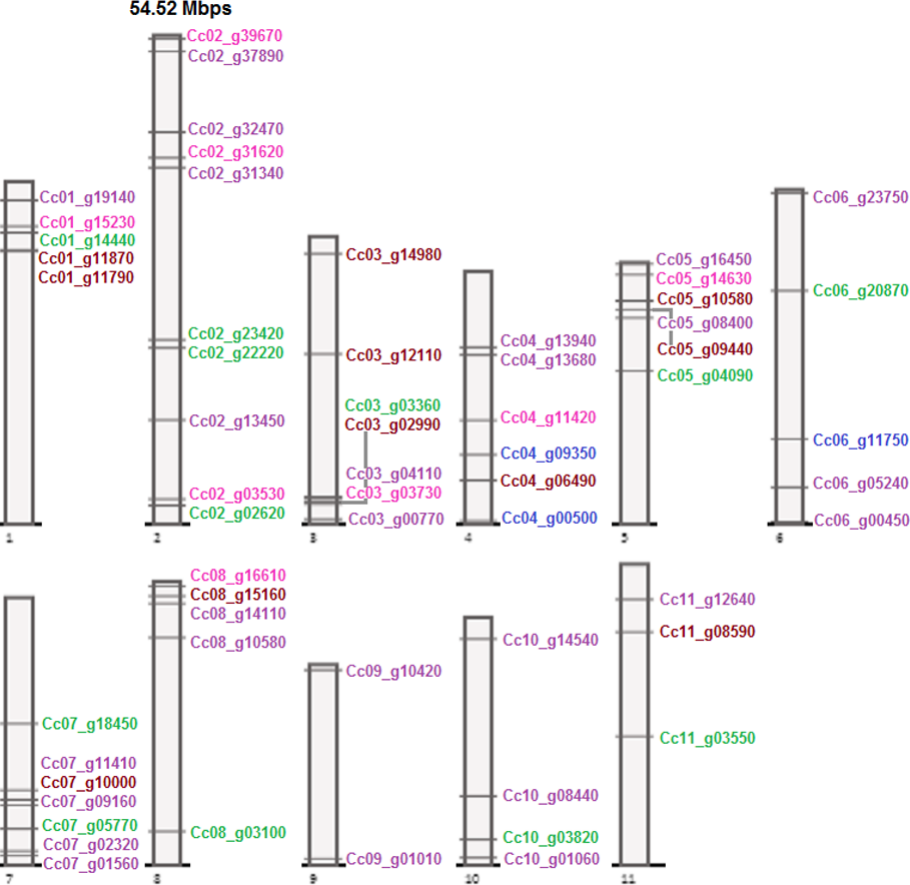
MPK, MKK, MEKK-like, Raf-like, and ZIK gene distribution on coffee chromosomes. Color code: maroon, MPK; blue, MKK; green, MEKK-like; purple, Raf-like; magenta, ZIK. Drawn to scale in reference to chromosome 2 labeled on top. Positions on the chromosomes ascending towards top.

Similarly for coffee, the random chromosomal distributions of genes on coffee chromosomes are represented in Fig. 9. MPK genes in coffee concentrated more on chromosome 3 and were also present on chromosomes 1, 4, 5, 7, 8, and 11. Coffee MKK genes were seen only on two chromosomes, 4 and 6. All chromosomes except 4 and 9 were seen to bear MEKK-like genes with larger number on chromosome 2. CcRaf-like genes were distributed on all chromosomes, the highest number being on chromosome 2 and 7. ZIK genes mostly concentrated on the chromosome 2 of coffee genome and were also present on chromosomes 1, 3, 4, 5, and 8. According to Holub’s gene clusters (Holub, 2001), no cluster was formed on coffee chromosomes but relaxed criteria suggests that two genes, Cc01_g11790 and Cc01_g11870, belonging to the MPK family formed one cluster on chromosome 1 of coffee.

Hence, it can easily be observed that the genes from all the three families of the MAPK cascade are uniformly distributed across the genome and occupy positions on all the chromosomes of these species. Among these species, number and positions of the MPK genes were almost conserved on the chromosomes of tomato, potato, and pepper; however, movements of the genes did occur on the chromosomes (Figs. 6–9, Table S9). To some extent this was also seen in coffee but the change in genome locations was greater in this case, as can be expected due to the early divergence of *Rubiaceae* from *Solanaceae* around 50 million years ago (Crepet, Nixon & Gandolfo, 2004; Gandolfo, Nixon & Crepet, 1998). Uniformity of distribution among genes, belonging to the MAPK cascade families and subfamilies in these species, was seen on chromosomes with respect to number of genes and presence or absence of genes on the respective chromosomes (Table S9).

Similar results were observed for MKK in terms of conservation; that is, the number and positions of most of these genes were conserved among chromosomes of tomato, potato, and pepper, while conservation in positions on coffee chromosomes in comparison to the *Solanaceae* species wasn’t observed (Figs. 6–9, Table S9).

In case of MAPKKK, more similarities in number and position of genes occurred between tomato and potato than with pepper for either one of them, while least with coffee (Figs. 6–9, Table S9).

Both tandem and segmental duplications are represented by the few clustered as well as random distribution of genes of the gene families as seen by the chromosomal locations of these genes. Even though, coffee shows similarity to *Solanaceae* species’ gene structures, coffee genes tend to have differences in chromosomal locations at a large extent with respect to *Solanaceae* species. Also, conservation in gene content was seen to be related to chromosomal position as observed for most genes of *Solanaceae*, the genes in the same or nearby clade in the phylogenetic trees tend to be present at adjacent chromosomal locations of the genomes. From an evolutionary perspective, the three families studied here developed more in *Solanaceae* than in *Rubiaceae* as can be seen by the larger number of genes belonging to these families in *Solanaceae* species compared to coffee from *Rubiaceae*. More importantly, the largest number of genes in the families of the MAPK cascade in potato elucidate the expansion of these families in potato over the course of evolution.

The members of these gene families need to be characterized in detail for evaluating their role in stress response and redundancy of their roles. This study will be helpful in designing future studies for gene expression analysis in these crop species with huge importance in agriculture and high impact on economy. Study of gene expression patterns will further help in developing the understanding about behavior of these genes under various environmental conditions leading to improvements in growth and cultivation, and hence the food quality as well as quantity.

## Conclusion

The three important families of the MAPK cascade reported here show a high extent of homology between the crops of *Solanaceae* and *Rubiaceae*. Various number of genes for each gene family were identified and characterized for various properties, phylogenetic relationship, gene structure and chromosomal location. Conservation, over the time of evolution, in the characteristics of these genes reflect their importance in basic plant processes. They also represent the occurrence of duplications in evolution. It was clearly observed that the MAPKKK family evolved to a greater extent in all the species. Also among these five crop species, all the three subject families of the MAPK cascade were found to have developed more in terms of number of paralogs in potato over the course of evolution. Conservation pattern of some paralog sequences suggests the basis for structural similarities which can lead to functional redundancy of these paralogs in the plant. Conservation was seen to be correlated in gene sequence, gene structure, and chromosomal location, and was in accordance with the clustering seen in the phylogenetic trees of the gene families. The reported genes will be studied for functional redundancy especially in terms of protein interactions in near future.

##  Supplemental Information

10.7717/peerj.3255/supp-1 Figure S1 Multiple sequence alignments of MPK gene proteins in the plant species marked at the regions containing conserved signature motif: (A) tomato; (B) potato; (C) eggplant; (D) pepper; (E) coffee.

10.7717/peerj.3255/supp-2 Figure S2 Multiple sequence alignments of MKK gene proteins in the plant species marked at the regions containing conserved signature motif: (A) tomato; (B) potato; (C) eggplant; (D) pepper; (E) coffee.

10.7717/peerj.3255/supp-3 Figure S3 Multiple sequence alignments of MEKK-like gene proteins in the plant species marked at the regions containing conserved signature motif: (A) tomato; (B) potato; (C) eggplant; (D) pepper; (E) coffee.

10.7717/peerj.3255/supp-4 Figure S4 Multiple sequence alignments of Raf-like gene proteins in the plant species marked at the regions containing conserved signature motif: (A) tomato; (B) potato; (C) eggplant; (D) pepper; (E) coffee.

10.7717/peerj.3255/supp-5 Figure S5 Multiple sequence alignments of ZIK gene proteins in the plant species marked at the regions containing conserved signature motif: (A) tomato; (B) potato; (C) eggplant; (D) pepper; (E) coffee.

10.7717/peerj.3255/supp-6 Figure S6 Distance matrix of the multiple sequence alignment of MPK protein sequences. if: Percentage shows the pairwise similarity among various sequences. Darker color shows higher similarity.

10.7717/peerj.3255/supp-7 Figure S7 Distance matrix of the multiple sequence alignment of MKK protein sequences Percentage shows the pairwise similarity among various sequences. Darker color shows higher similarity.

10.7717/peerj.3255/supp-8 Figure S8 Distance matrix of the multiple sequence alignment of MEKK-like protein sequences Percentage shows the pairwise similarity among various sequences. Darker color shows higher similarity.

10.7717/peerj.3255/supp-9 Figure S9 Distance matrix of the multiple sequence alignment of Raf-like protein sequences Percentage shows the pairwise similarity among various sequences. Darker color shows higher similarity.

10.7717/peerj.3255/supp-10 Figure S10 Distance matrix of the multiple sequence alignment of ZIK protein sequences Percentage shows the pairwise similarity among various sequences. Darker color shows higher similarity.

10.7717/peerj.3255/supp-11 Figure S11 Correlation plot analysis of the MPK genes in accordance with their distance-based UPGMA tree Correlation plot shows the variable percent of genetic content shared among the MPK genes on a scale from lowest depicted as red to highest depicted as green. Numbers at the top of the correlation plot represent the genes on the phylogenetic tree (numbers left to right = genes top to bottom).

10.7717/peerj.3255/supp-12 Figure S12 Correlation plot analysis of the MKK genes in accordance with their distance-based UPGMA tree Correlation plot shows the variable percent of genetic content shared among the MKK genes on a scale from lowest depicted as red to highest depicted as green. Numbers at the top of the correlation plot represent the genes on the phylogenetic tree (numbers left to right = genes top to bottom).

10.7717/peerj.3255/supp-13 Figure S13 Correlation plot analysis of the MEKK-like genes in accordance with their distance-based UPGMA tree Correlation plot shows the variable percent of genetic content shared among the MEKK-like genes on a scale from lowest depicted as red to highest depicted as green. Numbers at the top of the correlation plot represent the genes on the phylogenetic tree (numbers left to right = genes top to bottom).

10.7717/peerj.3255/supp-14 Figure S14 Correlation plot analysis of the Raf-like genes in accordance with their distance-based UPGMA tree Correlation plot shows the variable percent of genetic content shared among the Raf-like genes on a scale from lowest depicted as red to highest depicted as green. Numbers at the top of the correlation plot represent the genes on the phylogenetic tree (numbers left to right = genes top to bottom).

10.7717/peerj.3255/supp-15 Figure S15 Correlation plot analysis of the ZIK genes in accordance with their distance-based UPGMA tree Correlation plot shows the variable percent of genetic content shared among the ZIK genes on a scale from lowest depicted as red to highest depicted as green. Numbers at the top of the correlation plot represent the genes on the phylogenetic tree (numbers left to right = genes top to bottom).

10.7717/peerj.3255/supp-16 Figure S16 Schematic diagram of the exon-intron structure of the genes belonging to MPK family of the MAPK cascade: (A) Group D; (B) Group C; (C) Group A; (D) Group B. The direction of transcription is also shown. The gene structures are placed in accordance with the sequence order in phylogenetic tree. Color code: red, tomato; beige, potato; green, pepper; brown, coffee. Drawn to scale. Longest transcripts labeled from each clade.

10.7717/peerj.3255/supp-17 Figure S17 Schematic diagram of the exon-intron structure of the genes belonging to MKK family of the MAPK cascade: (A) Group A; (B) Group B; (C) Group C; (D) Group D. The direction of transcription is also shown. The gene structures are placed in accordance with the sequence order in phylogenetic tree. Color code: red, tomato; beige, potato; green, pepper; brown, coffee. Drawn to scale. Longest transcripts labeled from each clade.

10.7717/peerj.3255/supp-18 Figure S18 Schematic diagram of the exon-intron structure of the genes belonging to MEKK-like subfamily of the MAPK cascade: (A) Group A4; (B) Group A2; (C) Group A1; (D) Cluster 5; (E) Group A3. The direction of transcription is also shown. The gene structures are placed in accordance with the sequence order in phylogenetic tree. Color code: red, tomato; beige, potato; green, pepper; brown, coffee. Drawn to scale. Longest transcripts labeled from each clade.

10.7717/peerj.3255/supp-19 Figure S19 Schematic diagram of the exon-intron structure of the genes belonging to Raf-like subfamily of the MAPK cascade: (A) Group C5; (B) Group C6; (C) Group C4; (D) Group C3; (E) Group C7; (F) Group C2; (G) Group B4; (H) Group B1; (I) Group B2; (J) Group B3; (K) Group C1. The direction of transcription is also shown. The gene structures are placed in accordance with the sequence order in phylogenetic tree. Color code: red, tomato; beige, potato; green, pepper; brown, coffee. Drawn to scale. Longest transcripts labeled from each clade.

10.7717/peerj.3255/supp-20 Figure S20 Schematic diagram of the exon-intron structure of the genes belonging to ZIK subfamily of the MAPK cascade: (A) Cluster 2; (B) Cluster 4; (C) Cluster 5; (D) Cluster 3; (E) Cluster 1. The direction of transcription is also shown. The gene structures are placed in accordance with the sequence order in phylogenetic tree. Color code: red, tomato; beige, potato; green, pepper; brown, coffee. Drawn to scale. Longest transcripts labeled from each clade.

10.7717/peerj.3255/supp-21 Table S1 Characteristics of the database extracted sequences of MPK belonging to various plant species

10.7717/peerj.3255/supp-22 Table S2 Characteristics of the database extracted sequences of MKK belonging to various plant species

10.7717/peerj.3255/supp-23 Table S3 Characteristics of the database extracted sequences of MEKK-like belonging to various plant species

10.7717/peerj.3255/supp-24 Table S4 Characteristics of the database extracted sequences of Raf-like belonging to various plant species

10.7717/peerj.3255/supp-25 Table S5 Characteristics of the database extracted sequences of ZIK belonging to various plant species

10.7717/peerj.3255/supp-26 Table S6 Accession numbers of final sequences from plant species employed in the final datasets of the phylogenetic trees

10.7717/peerj.3255/supp-27 Table S7 Accession numbers of the sequences eliminated from the final dataset based on either minute changes in the signature motifs or partiality of sequences

10.7717/peerj.3255/supp-28 Table S8 Comparison of the number of introns possessed by genes of the plant species falling in various clusters of the phylogenetic trees

10.7717/peerj.3255/supp-29 Table S9 Family-wise comparison of the concentration of genes on the plant chromosomes

10.7717/peerj.3255/supp-30 Data S1 MPK sequence dataset

10.7717/peerj.3255/supp-31 Data S2 MKK sequence dataset

10.7717/peerj.3255/supp-32 Data S3 MEKK-like sequence dataset

10.7717/peerj.3255/supp-33 Data S4 Raf-like sequence dataset

10.7717/peerj.3255/supp-34 Data S5 ZIK sequence dataset

## Additional Information and Declarations

### Data Availability

The following information was supplied regarding data availability: The raw data has been supplied as a Supplementary File.
