# Supplementary figures and images for: In silico analysis reveals widespread presence of three gene families, MAPK, MAPKK and MAPKKK, of the MAPK cascade from crop plants of Solanaceae in comparison to the distantly-related syntenic species from Rubiaceae, coffee
Source: PeerJ. 2017 Jun 6;5:e3255. doi: 10.7717/peerj.3255 (PMC5463992; doi:10.7717/peerj.3255)

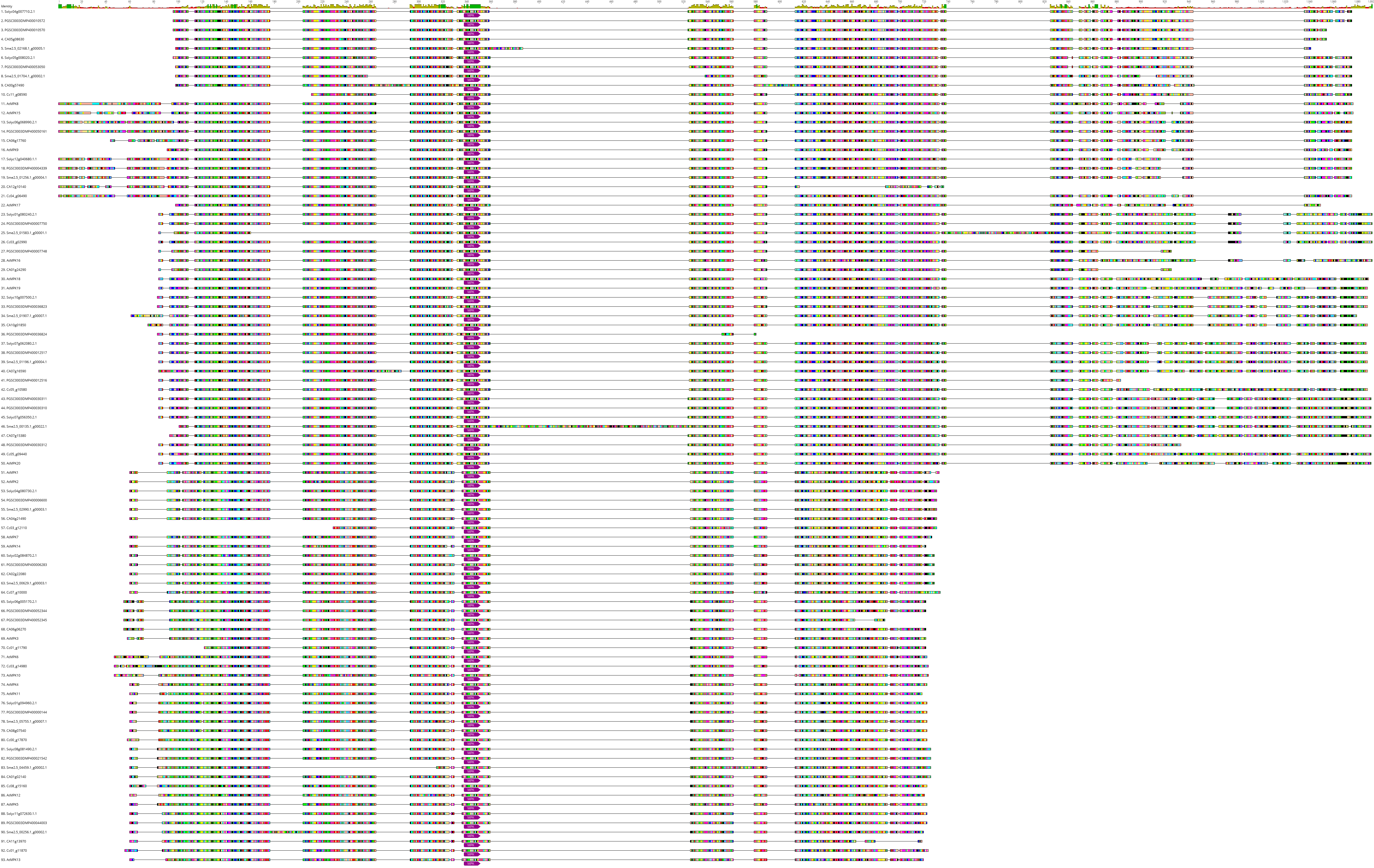

Supplement: Figure S1 — (A) tomato; (B) potato; (C) eggplant; (D) pepper; (E) coffee. [file peerj-05-3255-s001.png]

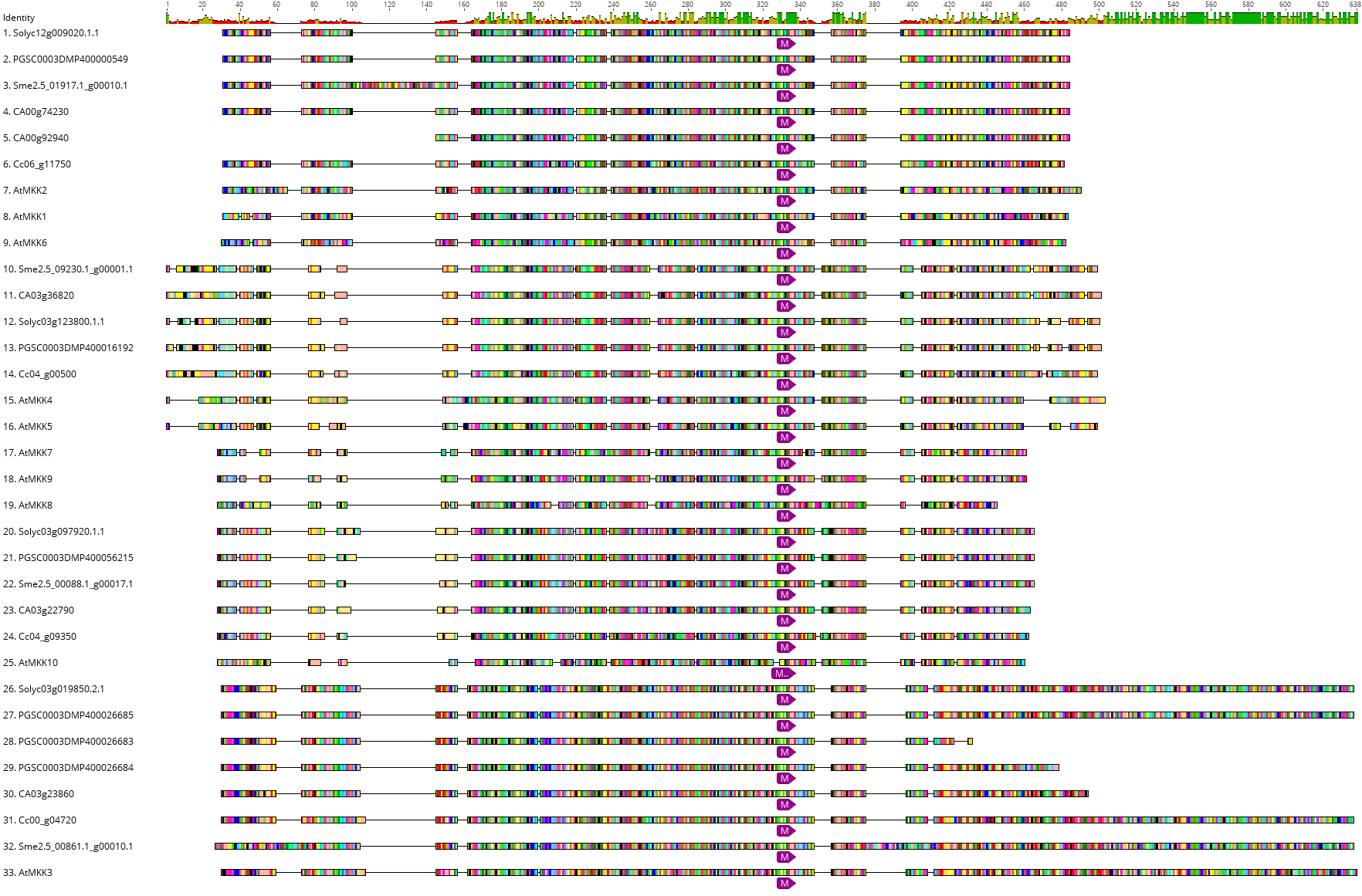

Supplement: Figure S2 — (A) tomato; (B) potato; (C) eggplant; (D) pepper; (E) coffee. [file peerj-05-3255-s002.png]

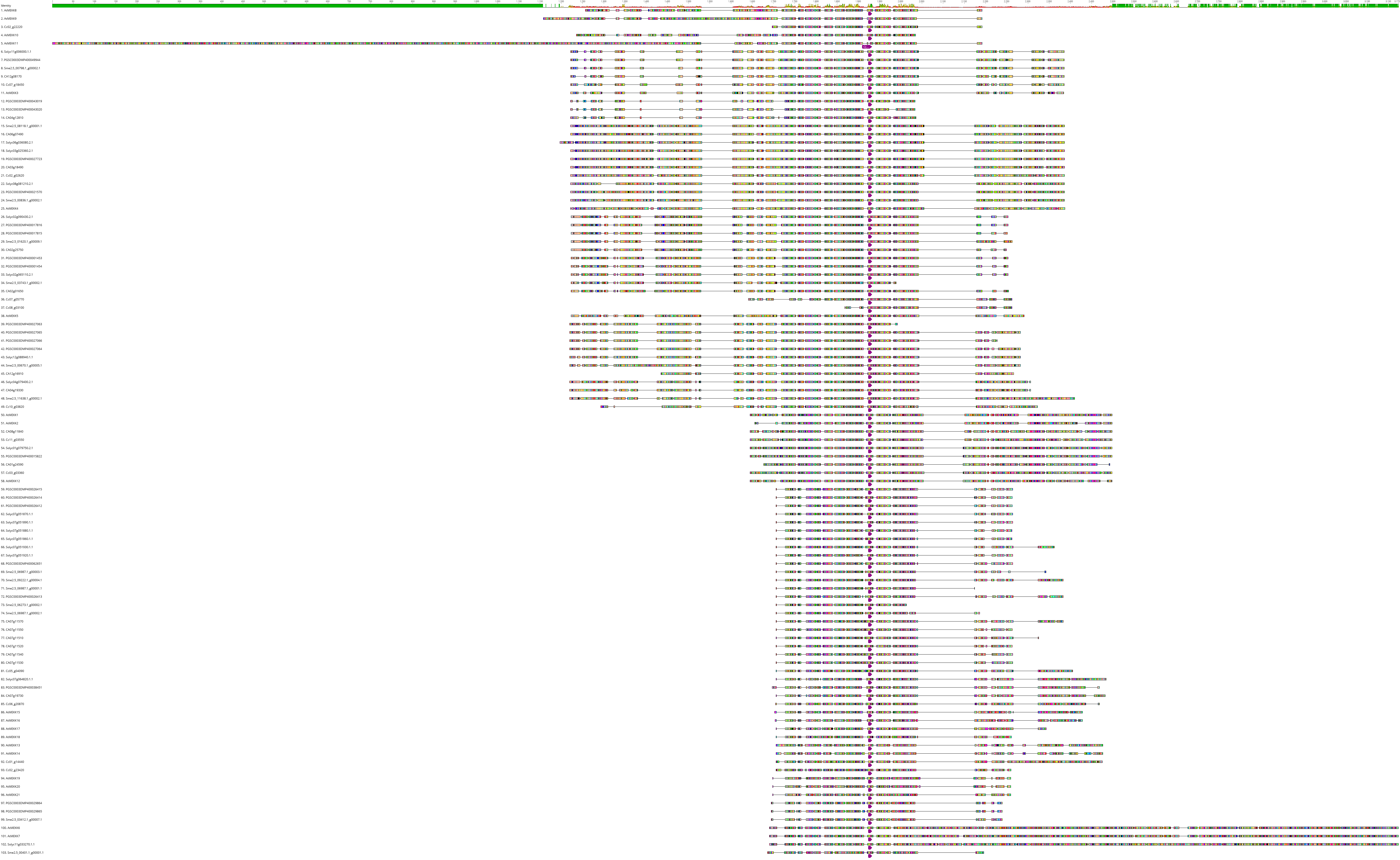

Supplement: Figure S3 — (A) tomato; (B) potato; (C) eggplant; (D) pepper; (E) coffee. [file peerj-05-3255-s003.png]

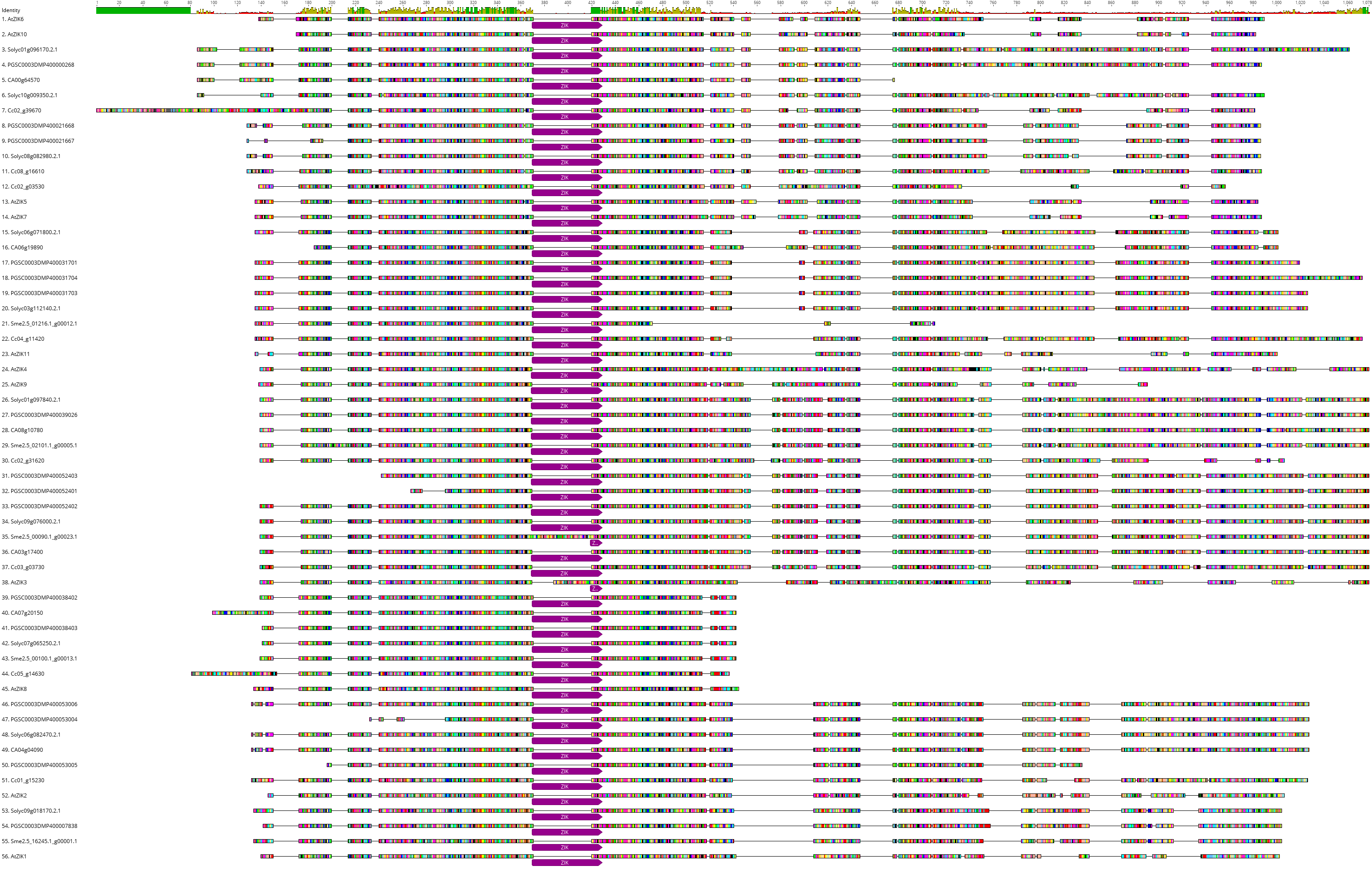

Supplement: Figure S5 — (A) tomato; (B) potato; (C) eggplant; (D) pepper; (E) coffee. [file peerj-05-3255-s005.png]

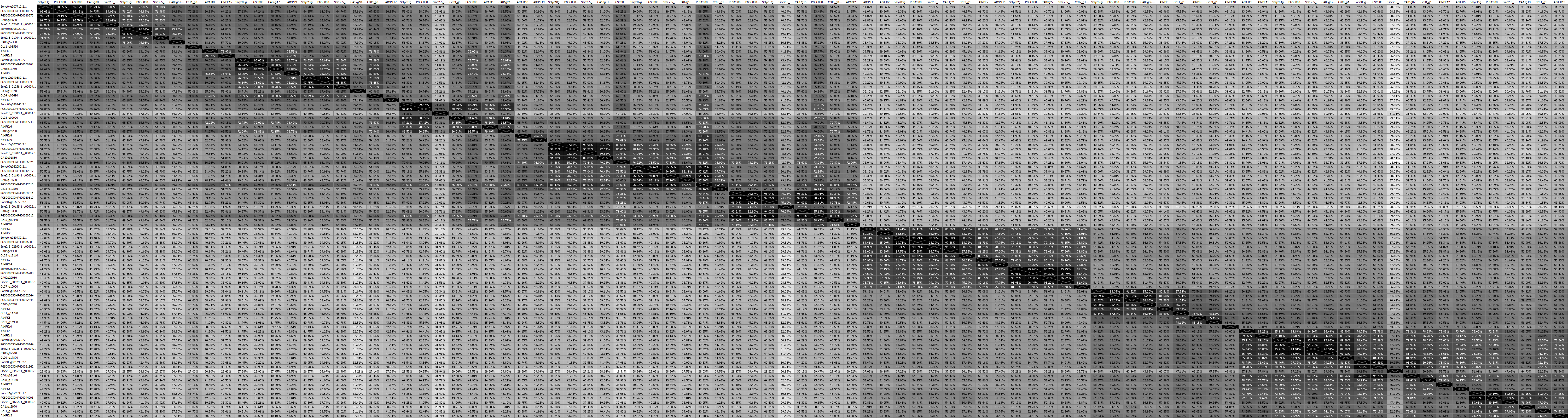

Supplement: Figure S6 — Percentage shows the pairwise similarity among various sequences. Darker color shows higher similarity. [file peerj-05-3255-s006.png]

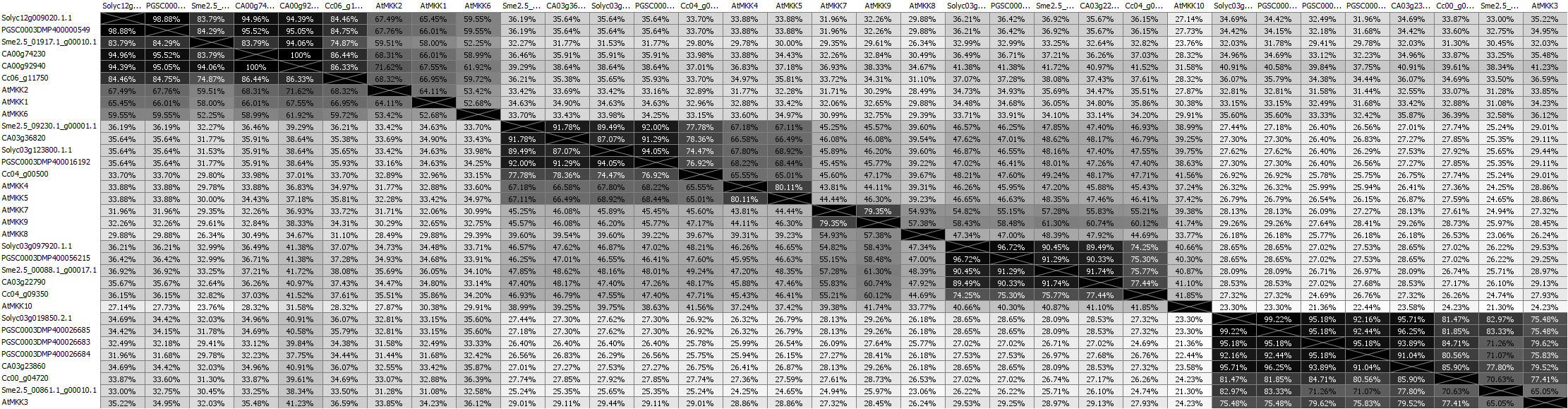

Supplement: Figure S7 — Percentage shows the pairwise similarity among various sequences. Darker color shows higher similarity. [file peerj-05-3255-s007.png]

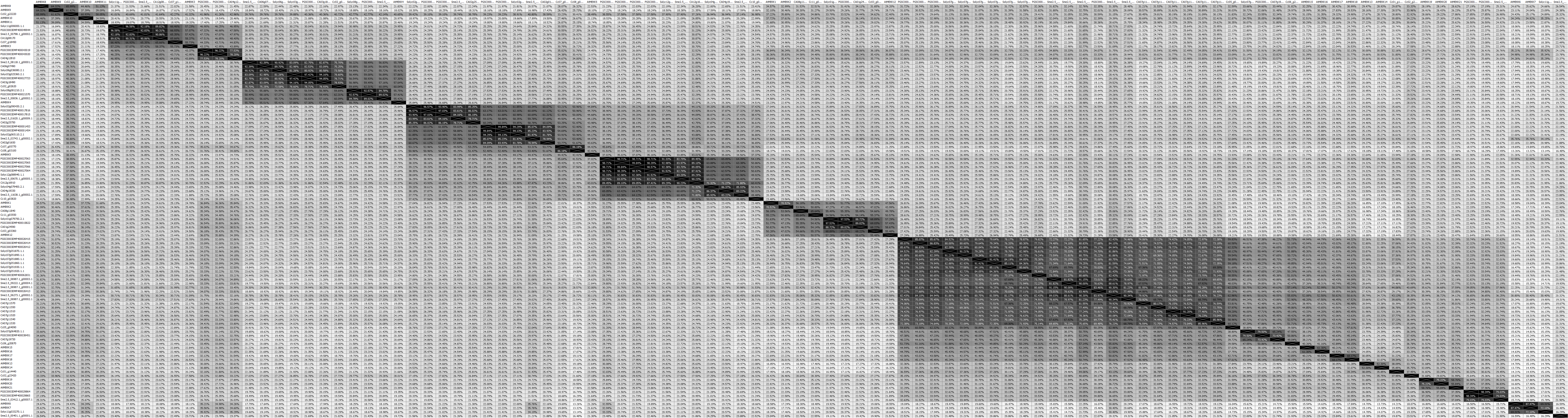

Supplement: Figure S8 — Percentage shows the pairwise similarity among various sequences. Darker color shows higher similarity. [file peerj-05-3255-s008.png]

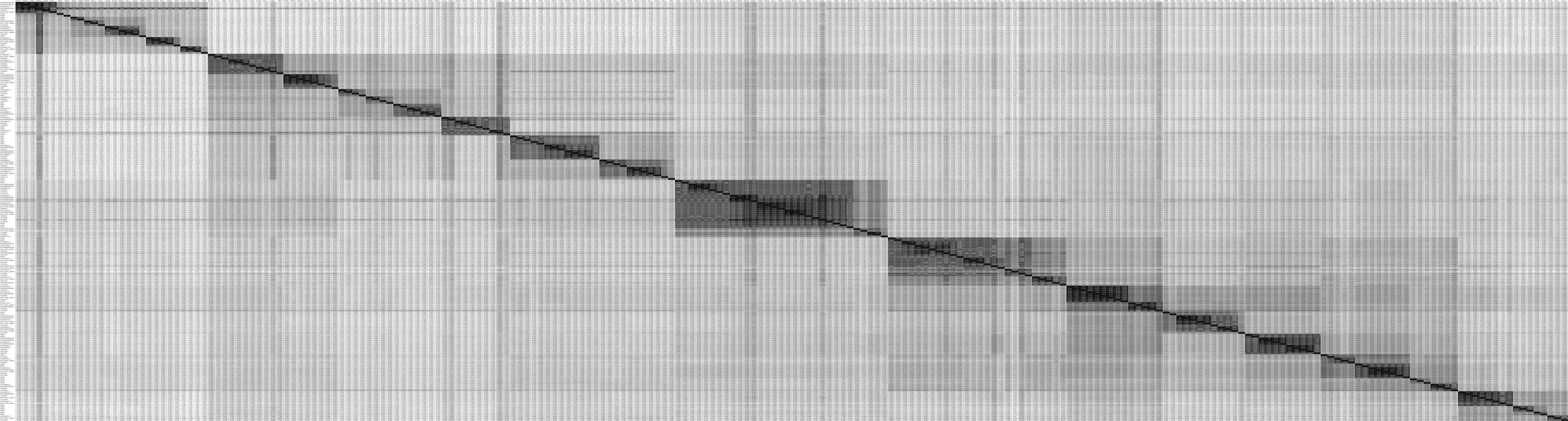

Supplement: Figure S9 — Percentage shows the pairwise similarity among various sequences. Darker color shows higher similarity. [file peerj-05-3255-s009.png]

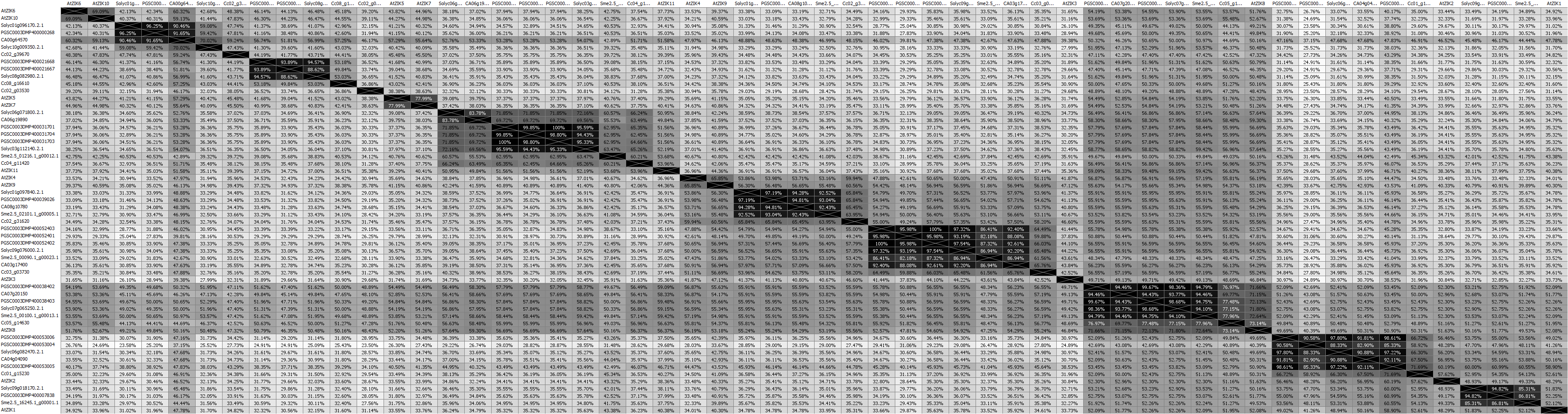

Supplement: Figure S10 — Percentage shows the pairwise similarity among various sequences. Darker color shows higher similarity. [file peerj-05-3255-s010.png]

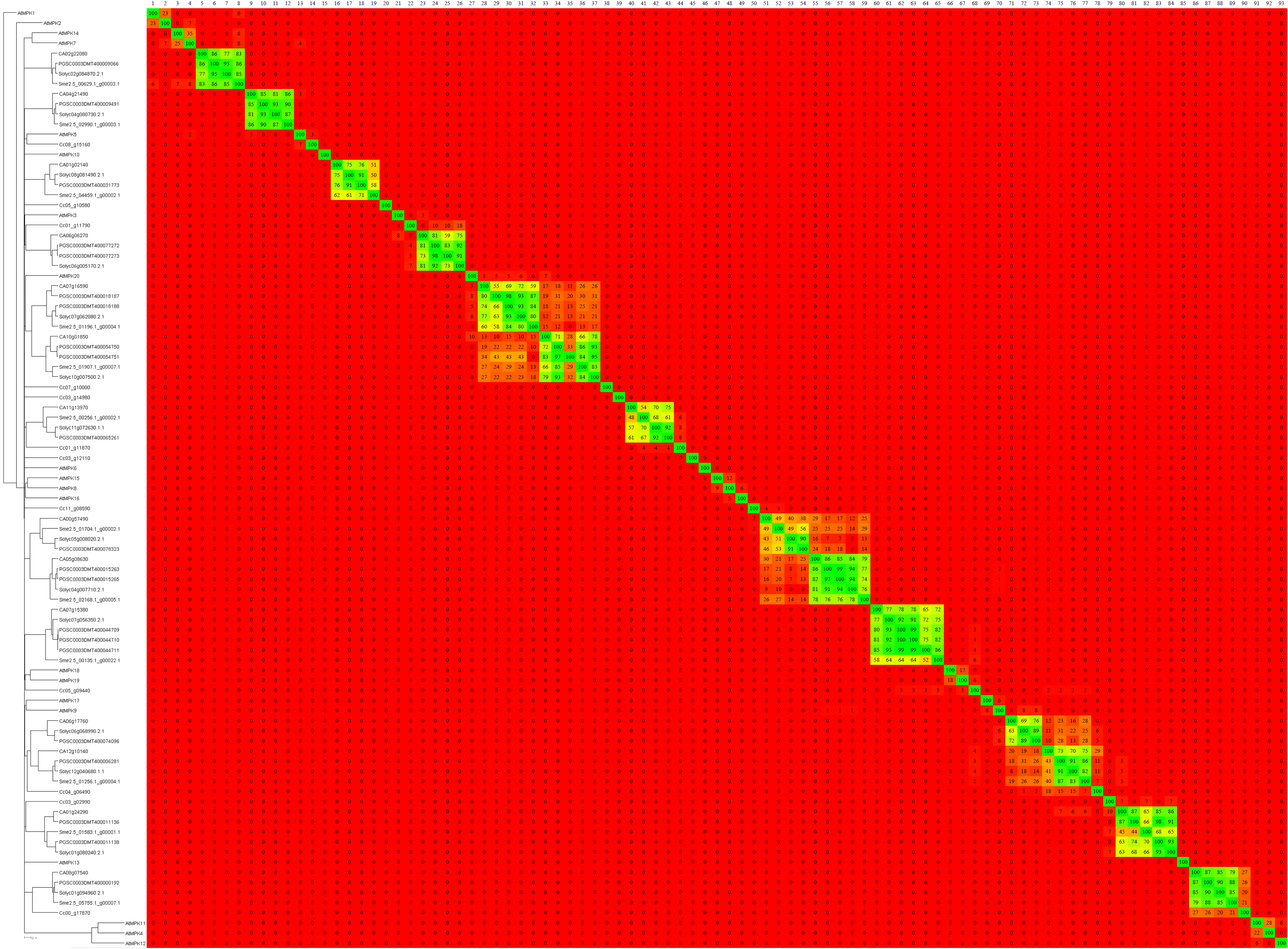

Supplement: Figure S11 — Correlation plot shows the variable percent of genetic content shared among the MPK genes on a scale from lowest depicted as red to highest depicted as green. Numbers at the top of the correlation plot represent the genes on the phylogenetic tree (numbers left to right = genes top to bottom). [file peerj-05-3255-s011.png]

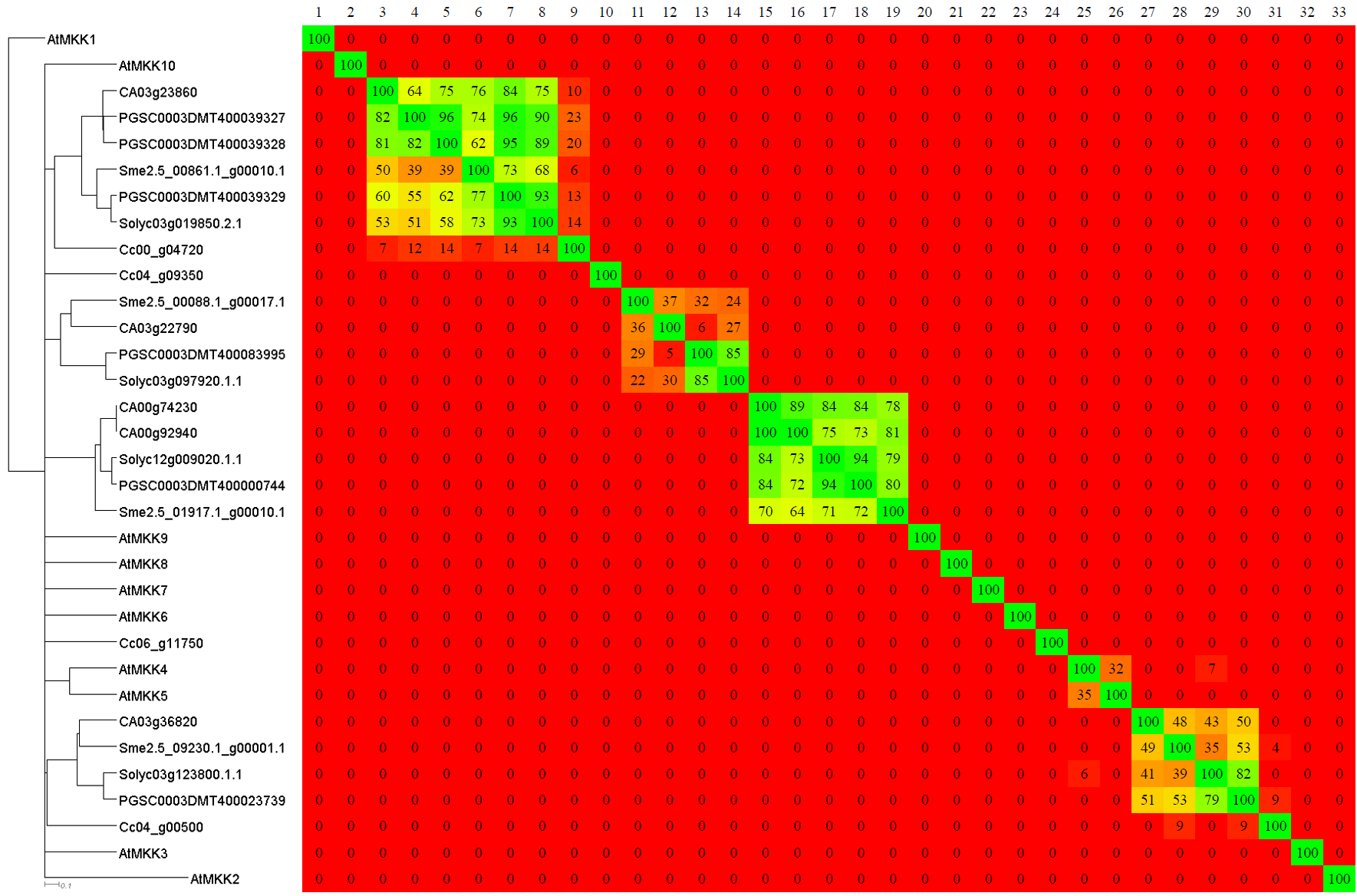

Supplement: Figure S12 — Correlation plot shows the variable percent of genetic content shared among the MKK genes on a scale from lowest depicted as red to highest depicted as green. Numbers at the top of the correlation plot represent the genes on the phylogenetic tree (numbers left to right = genes top to bottom). [file peerj-05-3255-s012.png]

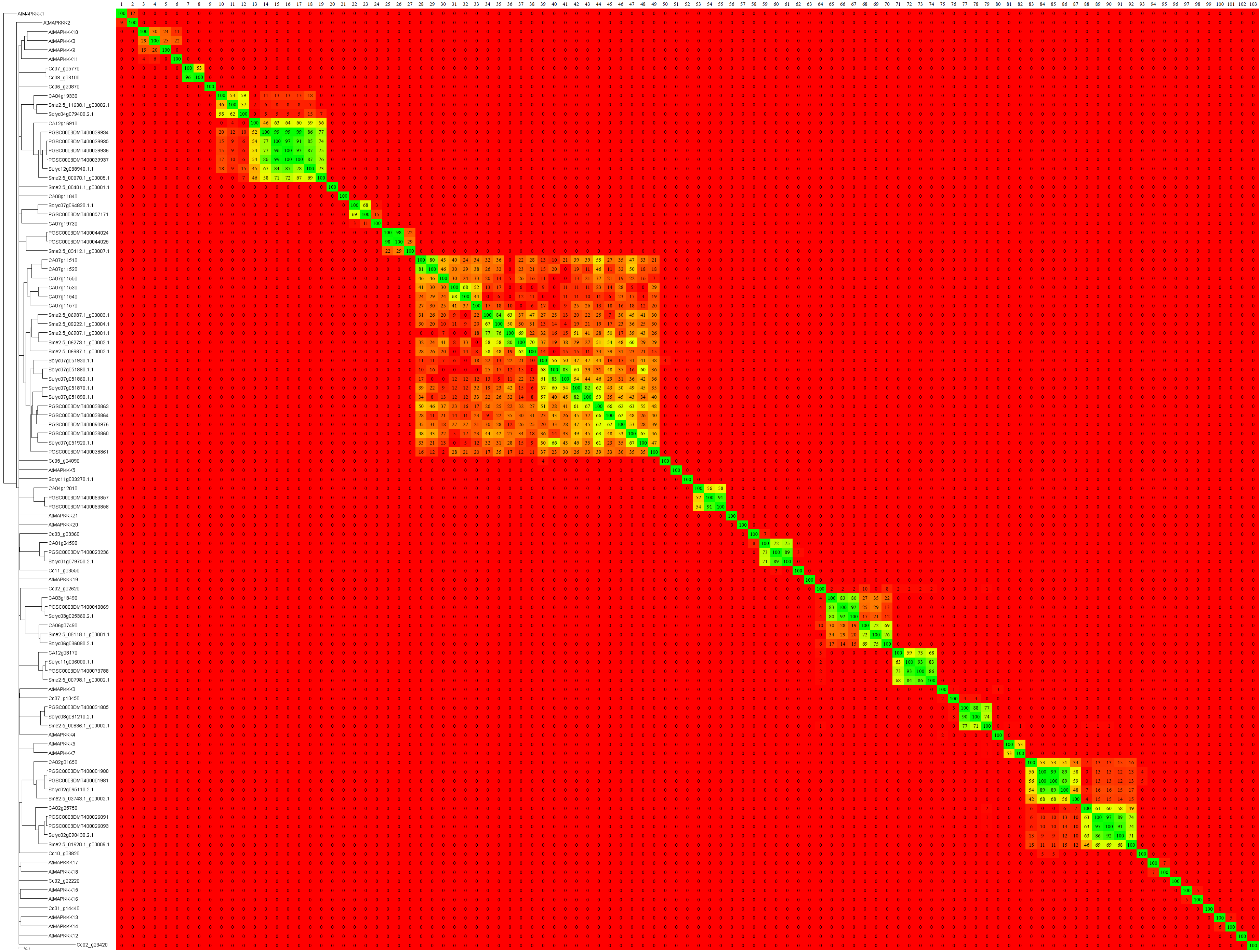

Supplement: Figure S13 — Correlation plot shows the variable percent of genetic content shared among the MEKK-like genes on a scale from lowest depicted as red to highest depicted as green. Numbers at the top of the correlation plot represent the genes on the phylogenetic tree (numbers left to right = genes top to bottom). [file peerj-05-3255-s013.png]

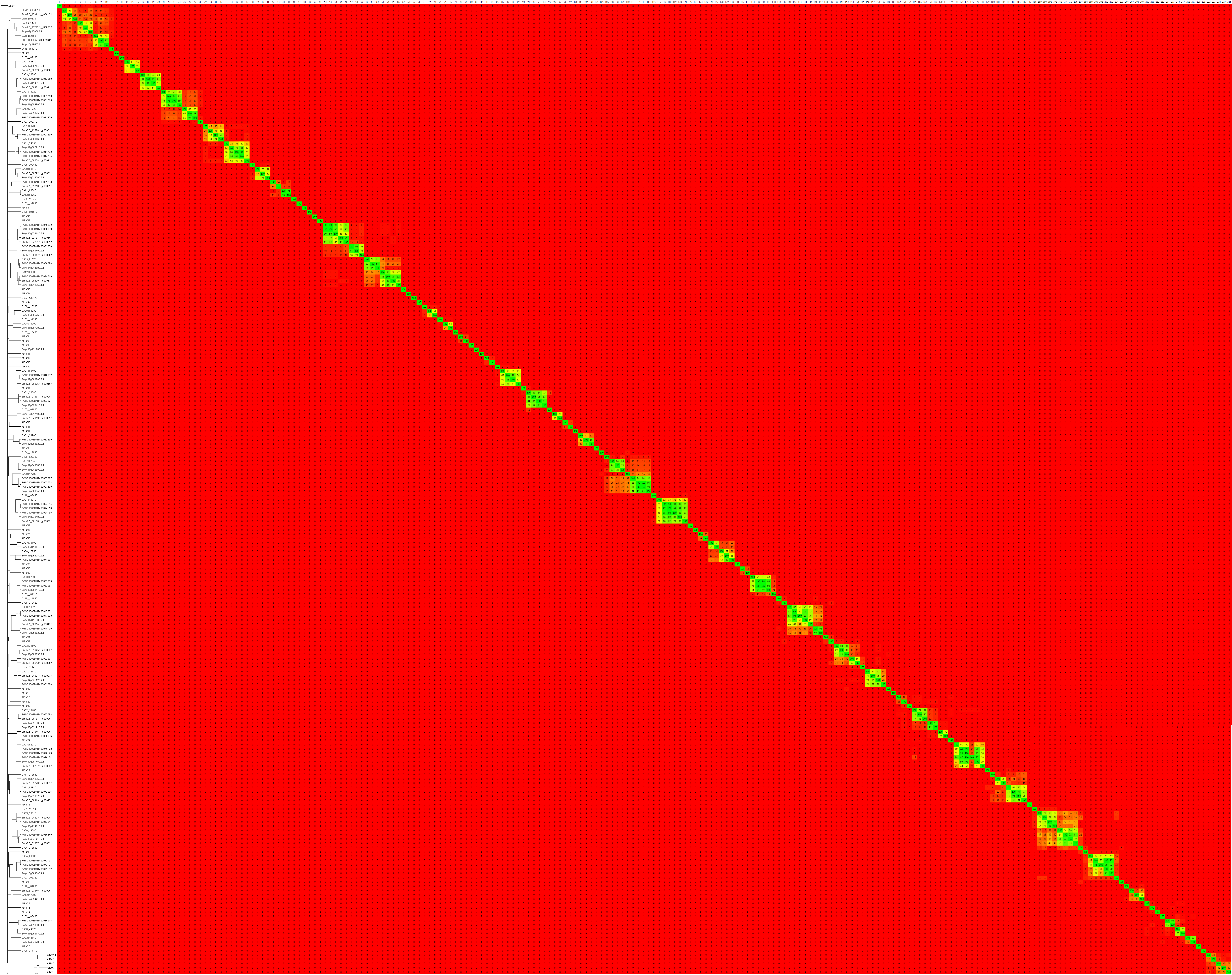

Supplement: Figure S14 — Correlation plot shows the variable percent of genetic content shared among the Raf-like genes on a scale from lowest depicted as red to highest depicted as green. Numbers at the top of the correlation plot represent the genes on the phylogenetic tree (numbers left to right = genes top to bottom). [file peerj-05-3255-s014.png]

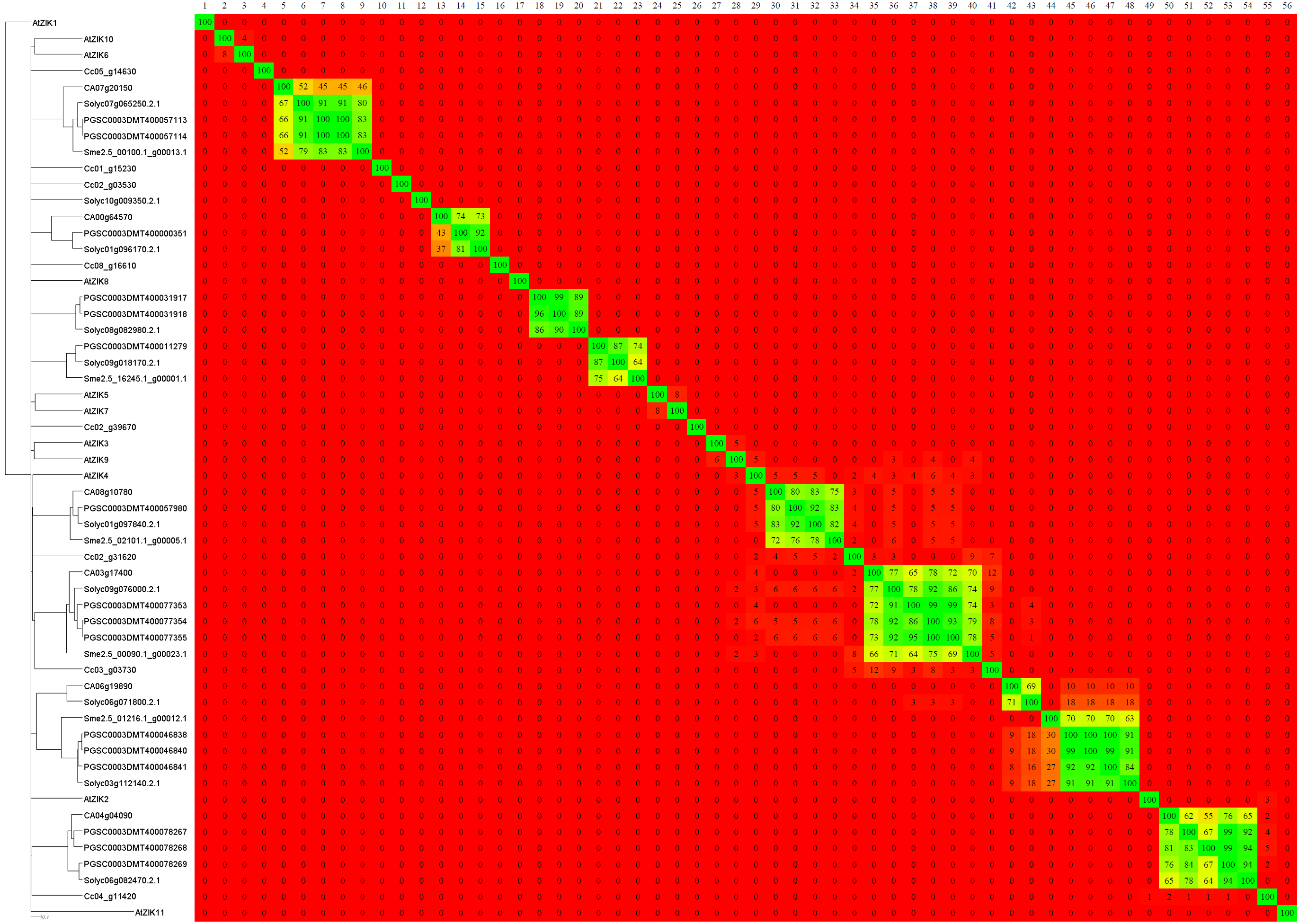

Supplement: Figure S15 — Correlation plot shows the variable percent of genetic content shared among the ZIK genes on a scale from lowest depicted as red to highest depicted as green. Numbers at the top of the correlation plot represent the genes on the phylogenetic tree (numbers left to right = genes top to bottom). [file peerj-05-3255-s015.png]

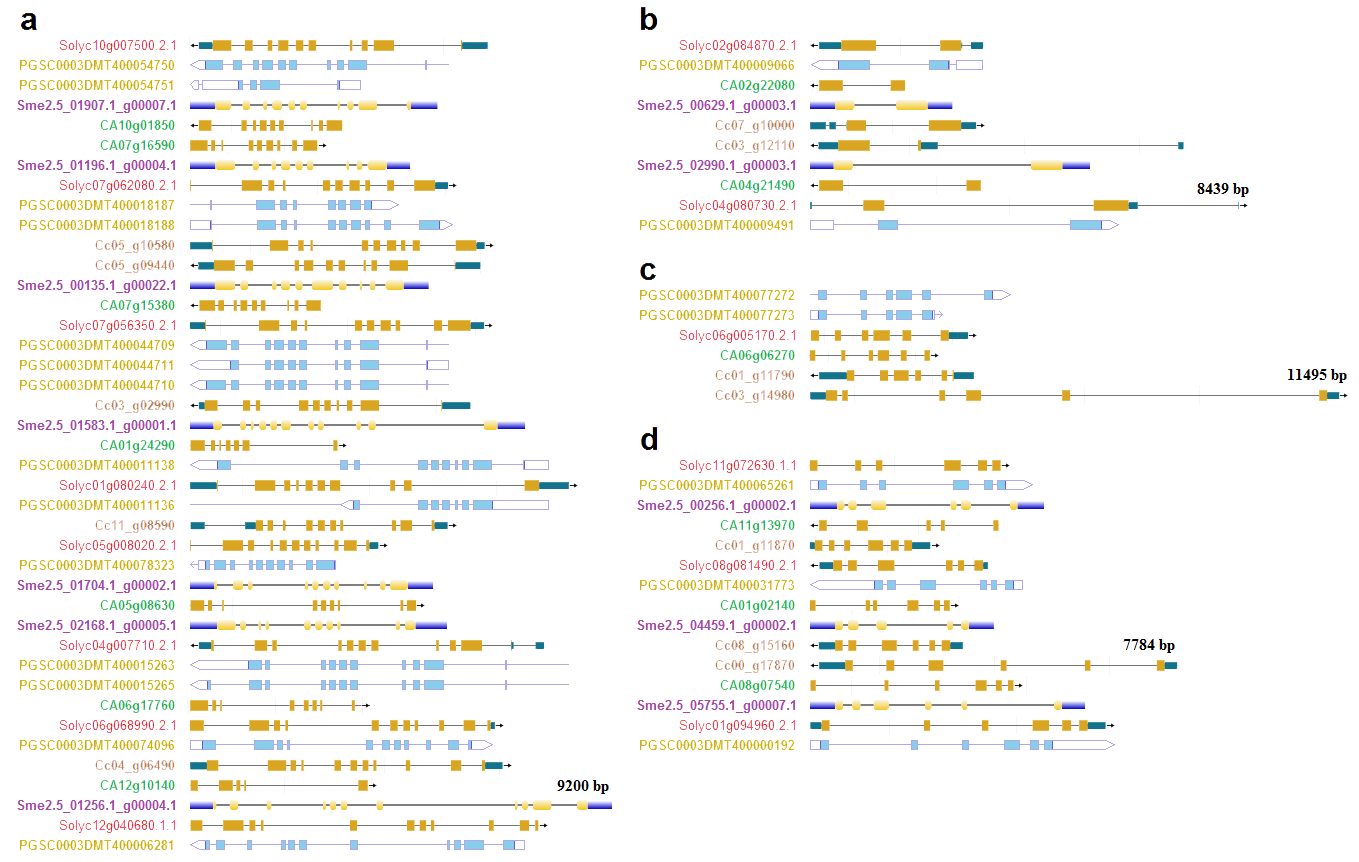

Supplement: Figure S16 — (A) Group D; (B) Group C; (C) Group A; (D) Group B. The direction of transcription is also shown. The gene structures are placed in accordance with the sequence order in phylogenetic tree. Color code: red, tomato; beige, potato; green, pepper; brown, coffee. Drawn to scale. Longest transcripts labeled from each clade. [file peerj-05-3255-s016.png]

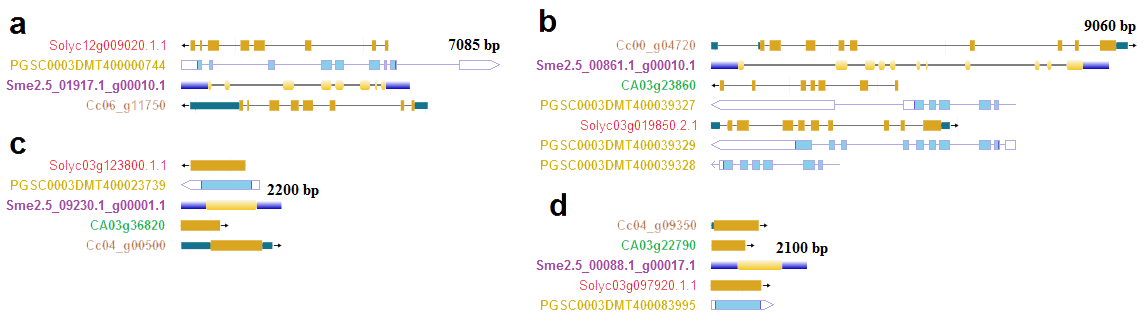

Supplement: Figure S17 — (A) Group A; (B) Group B; (C) Group C; (D) Group D. The direction of transcription is also shown. The gene structures are placed in accordance with the sequence order in phylogenetic tree. Color code: red, tomato; beige, potato; green, pepper; brown, coffee. Drawn to scale. Longest transcripts labeled from each clade. [file peerj-05-3255-s017.png]

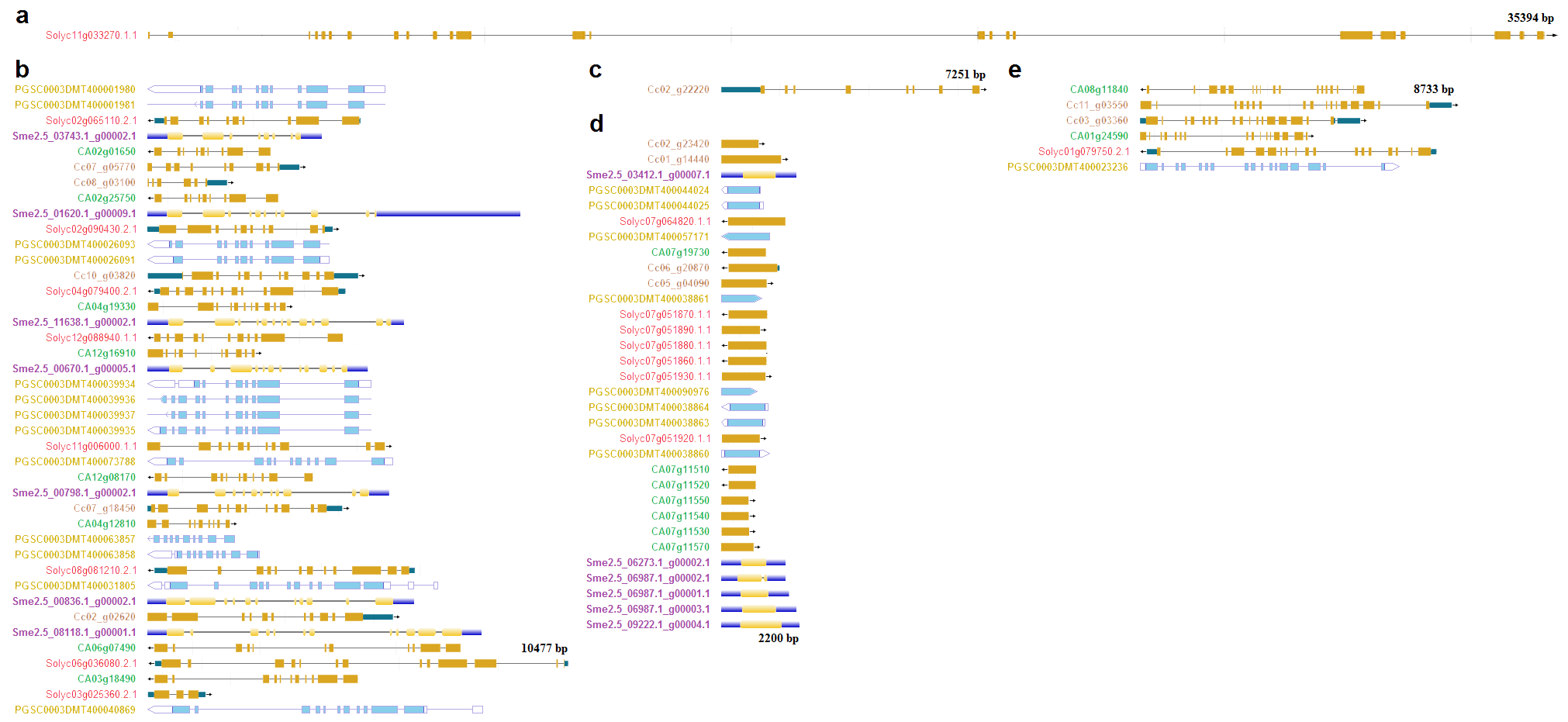

Supplement: Figure S18 — (A) Group A4; (B) Group A2; (C) Group A1; (D) Cluster 5; (E) Group A3. The direction of transcription is also shown. The gene structures are placed in accordance with the sequence order in phylogenetic tree. Color code: red, tomato; beige, potato; green, pepper; brown, coffee. Drawn to scale. Longest transcripts labeled from each clade. [file peerj-05-3255-s018.png]

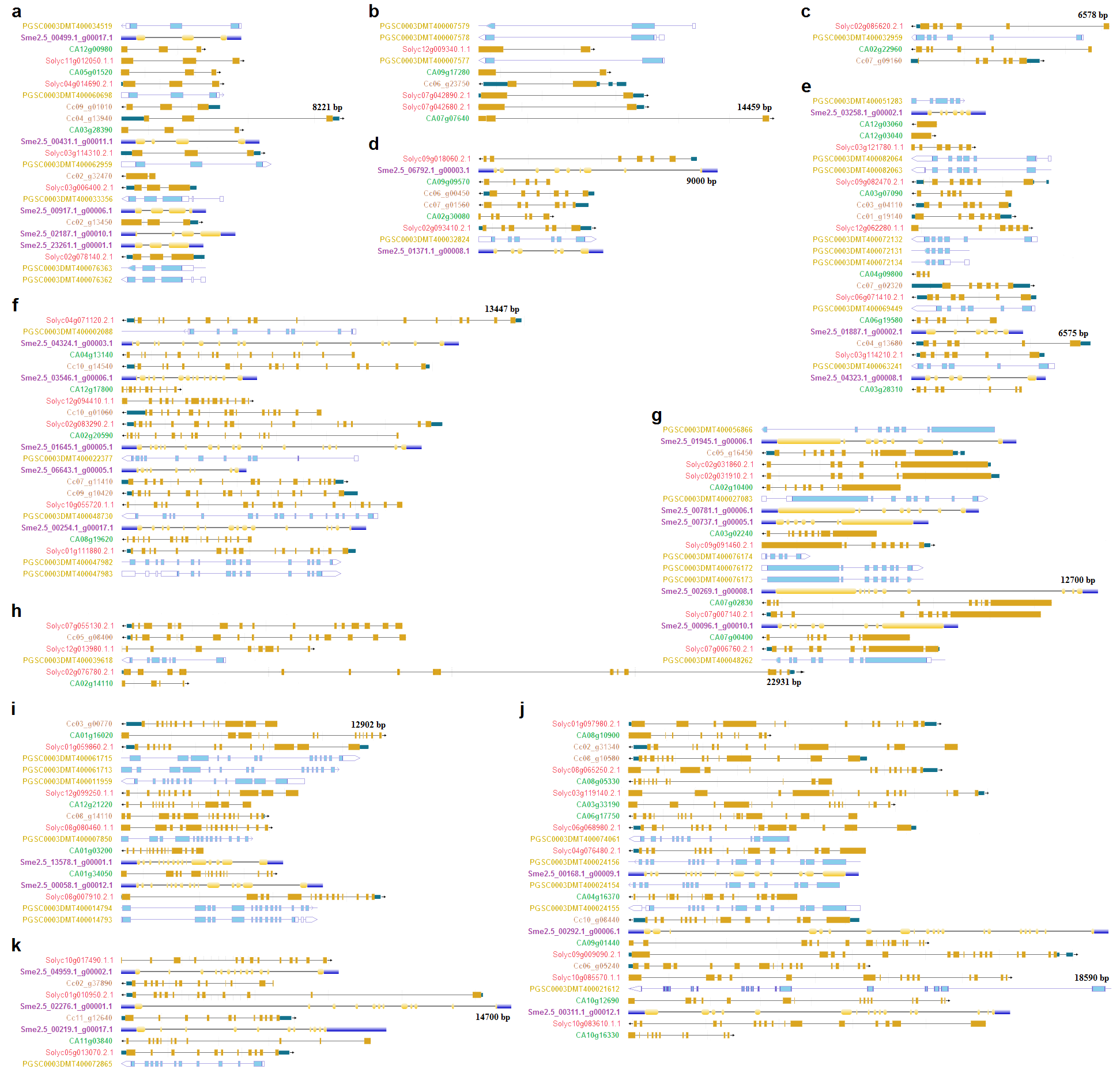

Supplement: Figure S19 — (A) Group C5; (B) Group C6; (C) Group C4; (D) Group C3; (E) Group C7; (F) Group C2; (G) Group B4; (H) Group B1; (I) Group B2; (J) Group B3; (K) Group C1. The direction of transcription is also shown. The gene structures are placed in accordance with the sequence order in phylogenetic tree. Color code: red, tomato; beige, potato; green, pepper; brown, coffee. Drawn to scale. Longest transcripts labeled from each clade. [file peerj-05-3255-s019.png]

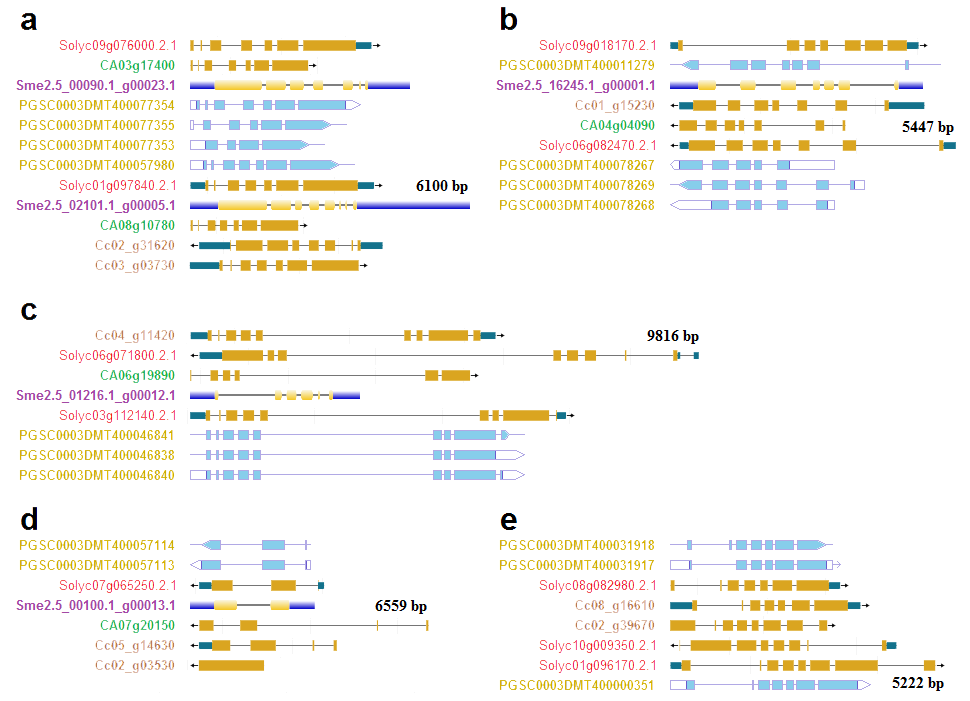

Supplement: Figure S20 — (A) Cluster 2; (B) Cluster 4; (C) Cluster 5; (D) Cluster 3; (E) Cluster 1. The direction of transcription is also shown. The gene structures are placed in accordance with the sequence order in phylogenetic tree. Color code: red, tomato; beige, potato; green, pepper; brown, coffee. Drawn to scale. Longest transcripts labeled from each clade. [file peerj-05-3255-s020.png]
